# 2D Semiconductor Nanomaterials and Heterostructures: Controlled Synthesis and Functional Applications

**DOI:** 10.1186/s11671-021-03551-w

**Published:** 2021-05-25

**Authors:** Hongyan Xu, Mohammad Karbalaei Akbari, Serge Zhuiykov

**Affiliations:** 1 School of Materials Science and Engineering, North University of China, Taiyuan, 030051 People’s Republic of China; 2 Centre for Environmental and Energy Research, Ghent University Global Campus, 119-5 Songdomunhwa-ro, Yeonsu-gu, Incheon, 21985 South Korea; 3 Department of Solid State Science, Faculty of Science, Ghent University, Krijgslaan 281/S1, 9000 Ghent, Belgium

**Keywords:** 2D semiconductors, Heterostructures, Synthesis, Atomic layer deposition

## Abstract

Two-dimensional (2D) semiconductors beyond graphene represent the thinnest stable known nanomaterials. Rapid growth of their family and applications during the last decade of the twenty-first century have brought unprecedented opportunities to the advanced nano- and opto-electronic technologies. In this article, we review the latest progress in findings on the developed 2D nanomaterials. Advanced synthesis techniques of these 2D nanomaterials and heterostructures were summarized and their novel applications were discussed. The fabrication techniques include the *state-of-the-art* developments of the vapor-phase-based deposition methods and novel van der Waals (vdW) exfoliation approaches for fabrication both amorphous and crystalline 2D nanomaterials with a particular focus on the chemical vapor deposition (CVD), atomic layer deposition (ALD) of 2D semiconductors and their heterostructures as well as on vdW exfoliation of 2D surface oxide films of liquid metals.

## Introduction

**Fig. 1:**
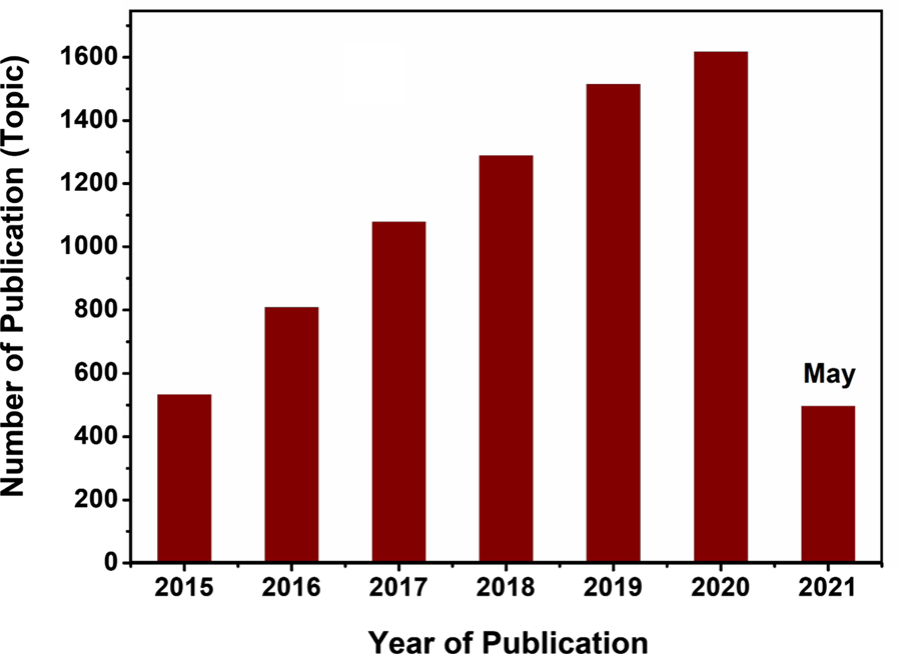
Dynamics of number of published articles for “*2D semiconductors*” based on Web of Knowledge™

Following the Noble price for graphene (2010) and subsequent advances in the development of 2D semiconductors beyond graphene, substantial growth of the independent scientific field of materials science based on 2D nanomaterials and their heterostructures has been witnessed during the second decade of the twenty-first century [1–4]. Very interesting physicochemical phenomena attributed to the electrons transfer and bandgap modulations have been observed in the structures of the reported 2D nanomaterials [5, 6]. Since graphene does not have bandgap, it cannot display semiconductor characteristics and tremendous attentions, and efforts were devoted to the development of other 2D nanomaterials analogous to graphene, including transition metal dichalcogenides (TMDCs), hexagonal boron nitride (*h*-BN), black phosphene, and transition metal oxides, etc. [7–16]. This family of 2D nanomaterials beyond graphene exhibited a wide spectrum of electronic characteristics covering a broad range of properties from metals to semi-metals and then from semiconductors to insulators [17–20]. Beside the broad range of electronic properties, other characteristics of 2D materials including their high-surface area, lack of dangling bonds, the nature of surface state, the distinguished spin-orbital coupling characteristics, and their quantum spin Hall effects orchestrate quite intriguing properties in the individual nanostructured 2D materials [21–25]. Consequently, the scientific communities’ interest to this type of 2D nanomaterials has been increasing exponentially particularly during the last decade due to their unprecedented semiconductor properties. In turn, this interest has been adequately reflected by the proportional exponential rise of the published articles dedicated to 2D semiconductors (Web of Knowledge™ database) presented in Fig. 1.

**Fig. 2:**
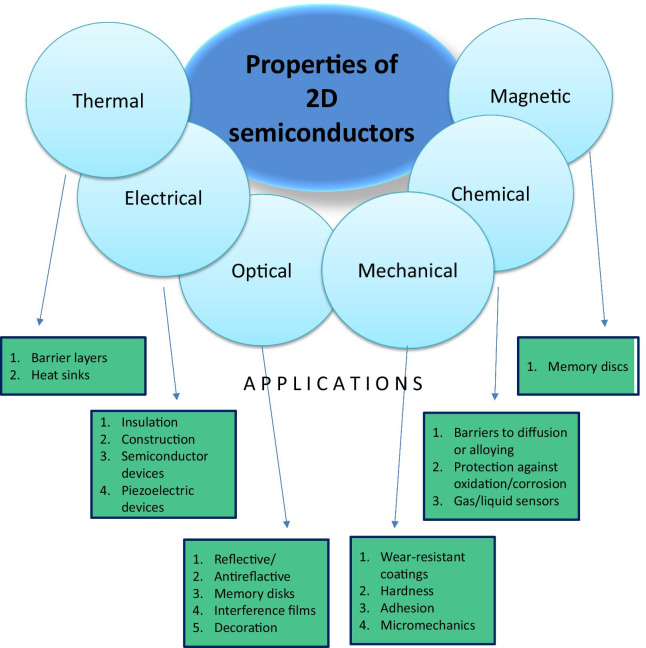
Properties of 2D semiconductors utilized in their typical applications

The early measurements of properties of 2D semiconductor nanomaterials revealed unique electronic characteristics, which were not comparable with the properties of their micro-structured and bulk counterparts. These distinguished features are related to the quantum confinement effects originated from the nanoscale low-dimensionality of 2D materials [26]. Therefore, these unique specifications of 2D materials provide excellent platforms to study their fundamental physics and chemistry. The first extensively investigated class of 2D nanomaterials is the layered TMDCs (*XY*_2_, where *X* = Mo, Ti, W and *Y* = S, Se and Te) [27–39]. The results obtained at this stage have confirmed that the dichalcogenide semiconductors have sizeable differences in the 2D and bulk conductivities (*σ*_1_/*σ*_2_ ~ 10^2^–10^3^) [40]. Furthermore, some of monolayer 2D materials, such as molybdenum disulfide (MoS_2_), is the direct bandgap semiconductor [41]. The obtained electronic characteristics of 2D TMDCs have vital implications to further development of the next generation of modern nano- and opto-electronic devices. These findings and other anisotropies arise from the presence of strong, interlayer *X*–*Y* bonding, which is in contrast to the weak vdW interactions between layers [42]. For example, TiS_2_ crystallizes in a hexagonal layered structure, where one hexagonally packed sheet of Ti atoms is sandwiched between two hexagonal sulfur sheets for each monolayer [43]. This specific layered configuration of atoms enabled the vdW delamination of layered 2D nanostructures. Consequently, most of the earlier reports were focused on the properties of 2D materials synthesized by mechanical and vdW exfoliation techniques. Owing to the ultra-thin nature of 2D films, they can be transparent, light and flexible and can also possess very interesting properties. These unique features paved the way towards the development of high-performance electrode materials with energy storage and conversion applications, like batteries, super-capacitors, and fuel cells [44]. For example, Fig. 2 summarizes the main properties of 2D semiconductors and heterostructures, which were utilized in their typical applications.

Noteworthy, the field of 2D nanostructured semiconductors stands up independently as important knowledge for development of modern high-performance nano- and opto-electronic instruments due to the latest technological advancements in nanofabrication [45]. The atomic-scale layered structure of 2D nanomaterials is the origin of their distinguished properties, where the precise tunability of materials properties is provided by the atomic level control of their dimensions. Thus, the conformal synthesis of ultra-thin 2D films with uniform thickness is highly desirable as a fundamental step towards the development of fabrication methods of advanced electronic instruments based on 2D nanomaterials. Specifically, for production of the wafer-scale ultra-thin films with precise and uniform thickness over the desired substrate, successful synthesis techniques for fabrication of instruments based on 2D semiconductors must be developed [46]. Furthermore, during the synthesis and fabrication stages, 2D nanomaterials could be decorated and may be in direct contact with various components of electronic units including electrical conductors, insulators and substrates with various geometrical features. It should also be taken into account that the hetero-interfaces between 2D nanomaterials and other components can fundamentally affect their properties. This is particularly related to the charge transfer mechanisms between 2D layers [47]. The unique features of hetero-structured 2D films allow them to be employed in many applications including hetero-structured electronics, optoelectronics, bio-sensing, environmental sensors, catalysis, wearable and flexible electronics, memristors, and synaptic devices [36, 44, 48–55]. Thus, the synthesis and fabrication techniques should in turn respond to the fabrication complexity of the functional instruments based on 2D nanomaterials. Moreover, the electronic properties of 2D materials are highly affected by the materials characteristics, including the physical and chemical structures, dimensions, number of layers, morphologies, orientations, structural phases, doping defects, amorphicity or crystallinity, and the grain boundaries in their structures [56]. The number of layers in 2D nanostructures has additional fundamental impact on the electronic structure and then the properties of 2D materials totally determine the final performance of the electronic devices based on the 2D films [57].

On the other hand, the crystallinity or amorphicity of 2D materials can also change their electronic properties. Determined by the synthesis methods and their conditions, 2D nanomaterials can either be *defects-free* (single crystal) or contain some impurities level or few grain boundaries. Therefore, the charge mobility through the interface scattering phenomenon is affected via the number of grain boundaries [58]. While a single crystalline structure has the highest charge mobility and low interface scattering, the amorphous 2D structures represent the highest insulating characteristics [58]. Most of the earlier synthesis methods of 2D nanomaterials were based on the mechanical exfoliation of 2D nanostructures. Due to weak vdW forces between the layered structures, the mechanical and chemical exfoliation can be effectively employed to extract the layered 2D nanostructures [59]. Thus, the mechanical exfoliation leads to separation of the high quality crystalline 2D structures from their host materials. However, mechanical exfoliation has already failed to synthesize the large-scale 2D nanofilms. Analogously, the liquid-phase exfoliation of 2D materials is another suitable approach for the low-cost, large-scale production of 2D materials. Nevertheless, the quality control and the size uniformity are the main challenges for synthesis of 2D films via this exfoliation technique [58]. On the contrary, the direct growth of 2D films via vapor-based deposition techniques was found to be one of the most reliable and applicable synthesis methods for fabrication of the high-quality wafer-scaled 2D nanofilms with precise dimensional control. For instance, CVD and ALD are two main vapor-based deposition techniques which offer distinguishable technical advantages for the large-area fabrication of nanostructured 2D semiconductors and their heterostructures [60, 61]. The precise control of thickness, chemical composition, crystallinity state, and even the percentage of doping elements and structural defects can be adequately achieved by careful manipulation of the growth parameters in CVD and ALD processes, respectively. In addition, apart from the conventional methods of synthesis of 2D films, other novel sources of 2D materials appeared just few years ago. For example, the surface oxide of liquid metals is in fact a unique source of high-quality natural 2D nanofilms with unprecedented properties arisen from the physical and chemical characteristics of liquid alloys [61].

Consequently, the review will discuss the latest findings on the 2D nanomaterials novel synthesis methods by focusing on ALD and CVD techniques and also on the vdW exfoliations of the 2D surface oxide of liquid metals. The growth mechanisms of 2D materials in the vapor phase deposition techniques are explained and the key challenges and opportunities in fabrication and deposition of 2D-based semiconductor materials are also discussed. Furthermore, the recently developed vdW exfoliation technique of the 2D natural surface oxide films of liquid metals is introduced. Finally, the *state-of-the-art* developed functional applications of ultra-thin 2D-based devices including their electronic and optoelectronic characteristics are presented and characterized in details.

## 2D Nanomaterials Synthesized by Vapor-Based Fabrication Techniques

The main focuses of researches working in the field of 2D semiconductor materials are on the synthesis and following characterization of 2D nanostructures. These 2D nanostructures belong to a broad range of inorganic, organic and polymeric materials with atomic and thin thickness. The synthesis strategies usually follow two main approaches, i.e., the *top-down* and *bottom-up* strategies. Therefore, the development of 2D semiconductors with controlled architectures and physical and chemical properties plays a key role in various applications and will open up new possibilities to the level that is significantly higher than today's commercial semiconductor technologies. Nonetheless, despite substantial efforts and unique scientific discoveries in the 2D semiconductors during last few years, most of them were obtained in the laboratories using quite simple exfoliation techniques, which cannot be used in industrial applications. One alternative represents the *top–down* method which typically involves the delamination and vdW exfoliation of layered materials and layered covalent organic frameworks (COFs). However, the bottleneck of this method is the extremely low yield of product and restacking of sheets during solidification. Another alternative is based on the *bottom-up* anisotropic assembly of inorganic, organic or polymeric precursors in a 2D manner. Although some success in the fabrication of 2D nanomaterials, the deep investigations of rationally designed 2D nanosheets with various functionalities in the electrochemical energy storage and conversion remain primitive. Thereby, the full explorations of the uniqueness of 2D nanomaterials with tailored thickness, high surface area, good conductivity and mechanical flexibility to accelerate ion diffusion and electron transport in various applications will be highly demanded. The following sections overview the fundamental of two main deposition techniques of 2D materials, i.e., CVD and ALD with focus on achievements during last few years.

### CVD of 2D Nanostructures

The vapor-phase-based direct growth of ultra-thin films is among the most reliable and applicable synthesis method for deposition of high-quality films with ultra-precise dimensional specifications [62]. Among various methods, CVD represents the first well-known successful technique for growth of ultra-thin 2D films on several different substrates. CVD offers a scalable and controllable approach for the growth of high-quality large area 2D films [63]. There are several distinguished specifications that make the CVD method a reliable technique for controllable deposition of 2D nanomaterials. The method is based on the step by step reaction of gaseous materials in vapor state on the surface of substrates. The CVD reactions are followed by the growth of solid-state thin films with few atomic layers thickness. Hence, the properties of 2D nanomaterials are highly dependent on the interfacial properties, geometrical features of substrate and structural phases of solid films. These properties could be carefully modulated by the precise adjustment of the CVD growth parameters. To understand the general mechanisms of CVD growth, it should be realized how the CVD parameters including temperature, pressure, substrate and precursors can affect the mass transfer, heat transfer and interfacial reactions on the surface. In this regard, a well-designed recipe controlling main deposition parameters during the process must be developed [64]. Moreover, it enables to control of level of doping in 2D nanofilms, which is vital capability in the design of semiconductor nanostructures with specific properties [64]. For example, it is possible to control the level of chalcogen and metal vacancies during the CVD process. The following key parameters of CVD process are highly dependent on the structural phases, morphology, and interfacial reactions.

#### Temperature

Temperature is one of the main fundamental parameters directly affecting several inter-connected factors during CVD film growth including the rate of chemical reactions of precursors, the flow rate of carrier gas, and finally, influencing the growth rate of CVD film [64]. Generally, high CVD temperature ensures deposition of the high-quality 2D CVD films. However, extremely high temperature has also disadvantages. Specifically, it can create high concentrations gradient and therefore causes unstable mass flow and transfer in the system [64]. It also directly affects the components saturation pressure in chamber and, consequently, alters the growth rate of thin films. The combined effects of temperature and pressure can be observed during CVD deposition of 2D TMDCs films. In this type of 2D nanostructures, the concentration of sulfur and selenium elements in the system is highly important to determine the final composition of 2D films. For instance, high gas pressure and concentration enabled the controllable synthesis of 2D TMDCs (MoS_2_, WS_2_, etc.). Generally, a high CVD temperature accompanied by the sufficient pressure facilitating the thermodynamically activated deposition mechanism, whereas a low CVD temperature usually leads to the kinetic growth process [64, 65]. The controlled growth mechanism finally results in the controlled growth of 2D TMDCs with the capability for adjustment of the properties and a number of fundamental layers.

#### Pressure

CVD is also known as variable pressure deposition technique, where the process pressure can be changed from atmospheric level to few millimeters. The most favourable condition for a controllable CVD process is achieved at the low concentration and high velocity of the mass feed. In this regard, the low pressure is always more reliable for uniform growth of ultra-thin 2D nanostructures at the wafer-scale deposition of TMDCs 2D nanostructures [66, 67]. The partial pressure of components can also directly affect the uniform layer-by-layer growth of 2D TMDCs films. Therefore, the growth of 2D MoS_2_ film by CVD technique is one of the most famous examples, where the fabrication of second fundamental layer over the initially grown film can only be initiated at the grain boundaries of the first deposited layer at the low pressure.

#### Substrate

**Fig. 3:**
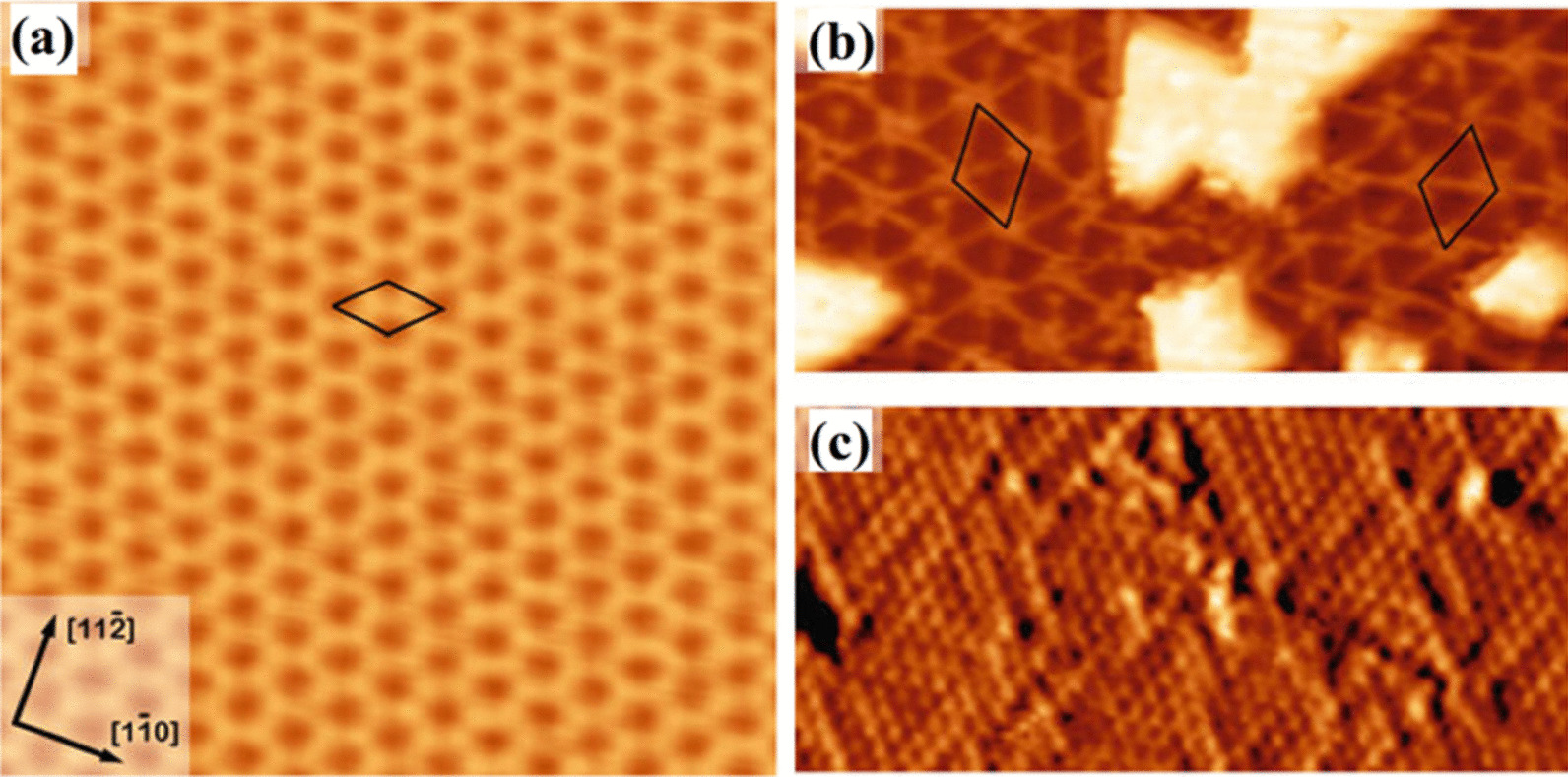
STM image of **a** the honeycomb Ti_2_O_3_ structure, and **b** pinwheel TiO monolayer grown on Au (111), and **c** atomically resolved STM image of pinwheel structures on Au (111). Reproduced with permission from [83]

The nature of substrate has fundamental impacts on the growth of 2D films and heterostructues. For example, metal substrate, Si, SiO_2_, mica, and polyimides are among the most commonly employed substrates for CVD deposition of 2D films [68, 69]. The role of substrates on the growth mechanism of 2D films determines their crystallinity, grains orientation, microstructures and properties. Specifically, 2D metal oxide films deposited on gold, nickel, copper, and silver substrates demonstrated the catalytic active properties [70–72]. Generally, in the case of metallic substrates, the preferential growth of 2D films with specific crystalline directions is technically dependent on the face-dependent binding energies between the metallic substrate and as-deposited TDMC film [73]. It also directly affects the morphology of as-grown nanostructures. In case of 2D metal oxides deposited on Au film, the bonding energies between Au and oxygen (O), and Au and metal (M) component of 2D oxide film determine the chemical bonding structures at the hetero-interface between Au and metal oxide film [73]. The preferential bonding between Au and metal atoms of oxide film is predicted, mostly resulting in the formation of Au-M–O interfaces [74–76]. The strong interaction between Au or other metallic substrates and as-deposited oxide thin films enabled the adaptive growth of 2D oxides from the crystalline structure of metal substrates [77–79]. It means that the growth pattern of the first metal oxide monolayer follows the crystalline pattern of metallic substrate. For instance, while the Au has the face-centered cubic (fcc) crystalline structure, its crystalline direction on the surface of Au substrate determines the crystalline orientation of 2D metal oxide film. Noteworthy, the unconstructed Au (111) consists of hexagonal lattices, while a reconstructed Au (111) plane shows a complex structure [80, 81]. It was observed that various deposited oxide films on the Au substrate lifted the herringbone reconstruction, which was caused by the strong interaction between Au (111) facet and the ultra-thin oxide film. Several different 2D metal oxides were grown on the Au substrates including TiO_*x*_, VO_*x*_, CoO, MoO_3_, MgO, ZnO, and WO_*x*_ [82]. One of the famous catalyst examples is 2D TiO_2_ grown on the surface of Au (111) substrate [83]. It was suggested that Ti atoms occupy the threefold hollow sites of Au lattice and O atoms position at the bridge sites of Ti atoms, then a honeycomb structure with stoichiometry of Ti_2_O_3_ is formed, shown in Fig. 3a [83]. Moreover, there is another mechanism of growth of 2D oxide films, where a M–O lattice superposes over the Au (111) surface and forms *Moiré* patterns resulting in the growth of different appearance of the pinwheel structures with TiO stoichiometry, as demonstrated in Fig. 3b and c, respectively [83]. The hexagonal *Moiré* pattern growth was commonly observed during the CVD of TiO, FeO [84], CoO [85], and ZnO [86] ultra-thin films on the Au (111) substrate.

**Fig. 4:**
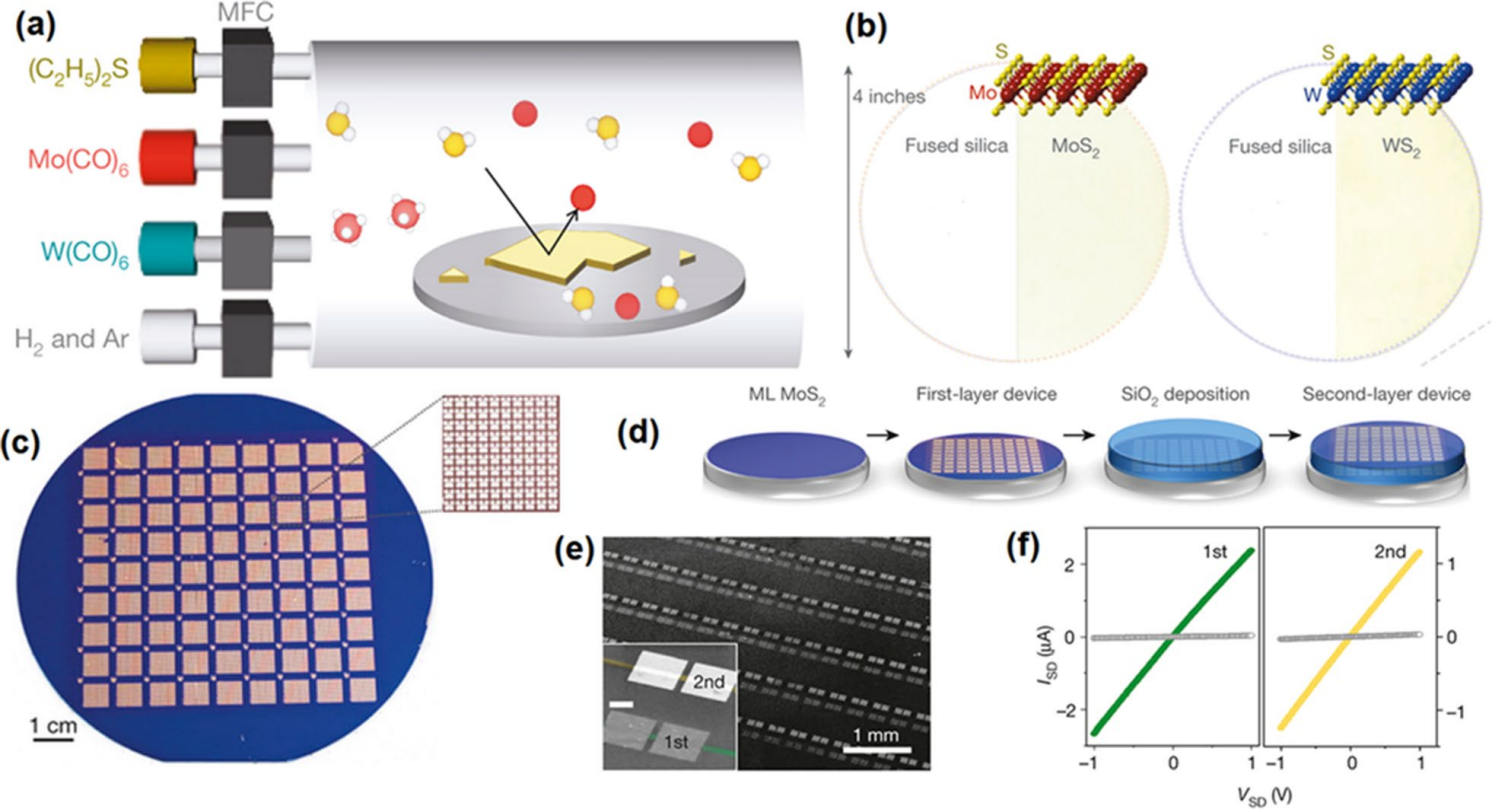
Wafer-scale MOCVD growth of continues MoS_2_ and WS_2_ film. **a** The graphical schematic of CVD device and the related precursors for MOCVD of 2D MoS_2_ and WS_2_ films. **c** The batch of 8100 FET devices based on 2D MoS_2_ film over Si/SiO_2_ wafer. The inset shows the enlarged image of one mm square containing 100 devices. **d** The sequential fabrication stage of stacked MoS_2_-based device. **e** The image demonstrates the MoS_2_ FET devices on first and second layers. **f** The IDS-VDS of two neighboring FET devices on two different layers. Reprinted with permission from [88]

MoS_2_ is a typical representative of the family of 2D TMDCS. So far it has been considered as one of the most favourable nanomaterials among all TMDCS as it has relatively small band gap of 1.29 eV and 1.90 eV for the bulk material and single layers, respectively [40, 87, 88]. Previously a single crystalline MoS_2_ 2D film was only mechanically exfoliated from its host bulk. Being a direct gap semiconductor, single layers of MoS_2_ offer the intriguing possibility for the realization of an inter-band tunnel field-effect transistor (FET), which is characterized by a turn-on sharper than the theoretical limit of 60 mV dec^−1^ for classical transistors, and consequently, smaller power dissipation. This feature has remained difficult to achieve in the case of silicon, an indirect gap semiconductor, because inter-band transitions there require phonons and recombination centers [87]. One of the interesting recent applications of 2D MoS_2_ nanocrystals is the transistor [87] and phototransistor [40] based on a single layer of MoS_2_ nanosheet. In addition, the successful large-area deposition of 2D films was achieved via modified CVD techniques including the metal–organic chemical vapor deposition (MOCVD), low-pressure chemical vapor deposition (LPCVD), inductively coupled plasma chemical vapor deposition (ICP-CVD), or other innovative methods which are developed and combined by other techniques. CVD is also well-known for its capability for wafer-scale deposition of 2D TMDC films. Figure 4 shows one of the interesting examples is the lager-area deposition of ultra-thin 2D MoS_2_ and WS_2_ films over 4-inch wafer [88]. In this CVD process, Mo(CO)_6_ and W(CO)_6_ and (C_2_H_5_)_2_S were employed as precursors in MOCVD technique, whereas the H_2_/Ar was used as a carrier gas (Fig. 4a, b) in MOCVD process [88]. It was demonstrated that 8100 high-performance FET units based on 2D MoS_2_ films were fabricated by using photolithography technique with the same back gate substrate (Fig. 4c). In the second configuration of the unit, external SiO_2_ dielectric film was deposited on the 2D MoS_2_ film to fabricate FET instruments (Fig. 4d). The electrical measurements of transistors confirmed the consistent performance of FET, which were selected from the different wafer parts. Hence the uniform and conformal growth conditions during the CVD process of 2D MoS_2_ and WS_2_ films were confirmed (Fig. 4f).

**Fig. 5:**
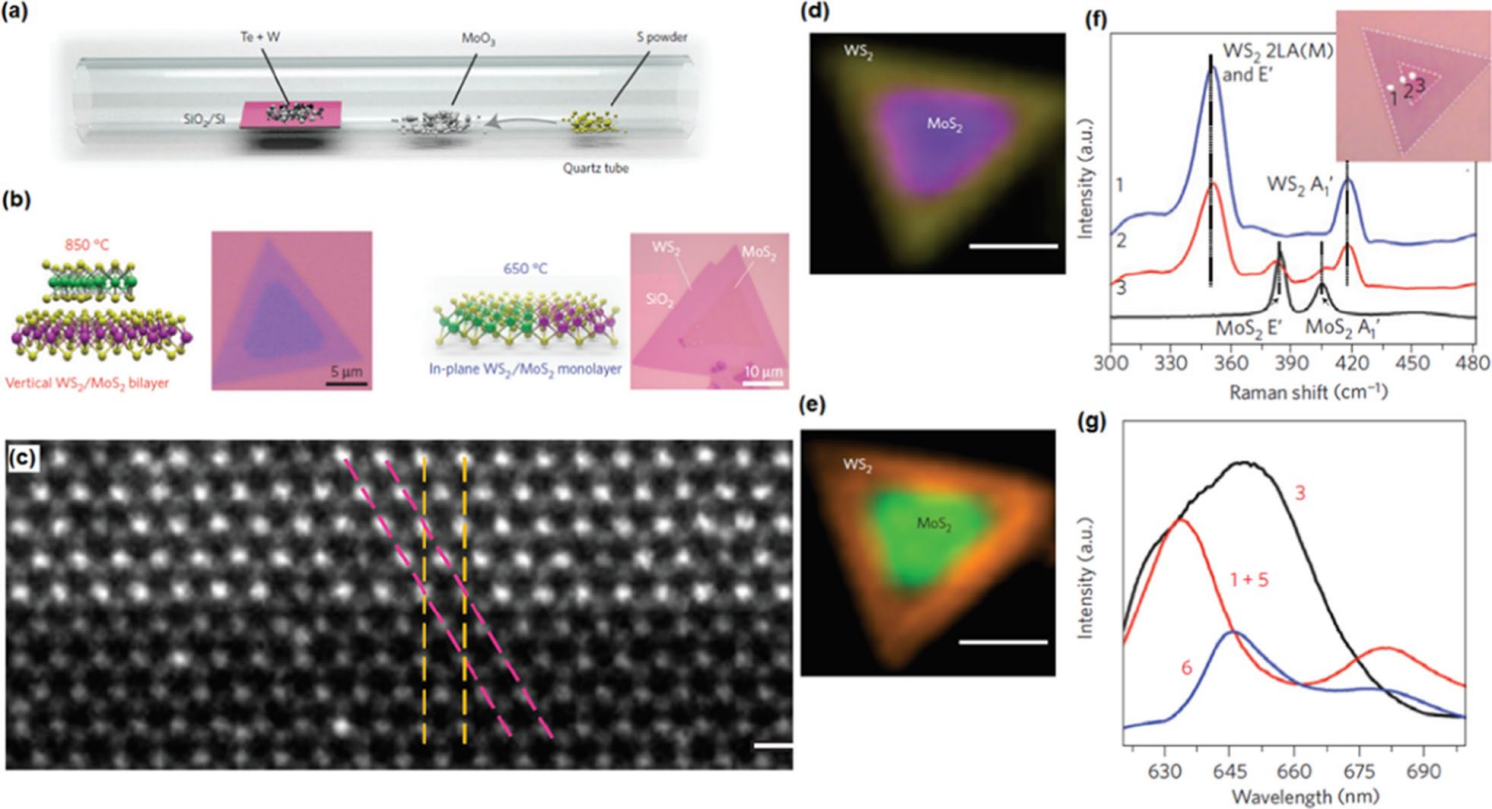
**a** Schematic of CVD growth of semiconductor/semiconductor lateral 2D heterostructures. **b** Atomic resolution contrast STEM images of in-plane interface between WS_2_ and MoS_2_. **c** The Raman intensity mapping of 2D heterostructured 2D film at 351 cm^−1^ (yellow) and 381 cm^−1^ (purple). **d** The combined PL intensity mapping at 630 nm (orange) and 680 nm (green). Reprinted and reproduced with permission from [89]

CVD has also established itself as versatile technique for fabrication various 2D heterostructures [89]. For example, CVD was employed for successful deposition of both lateral and vertical configurations. In the lateral style, the 2D nanofilms are attached together at the atomic level, while in the vertical structure, the interaction force between the layers has vdW nature. Thus, in the lateral 2D heterostructures, a strict lattice matching is highly necessary for development of the 2D hetero-interfaces, as was reported for MoS_2_/WS_2_ heterostructures [89]. One-step simple deposition of MoS_2_/WS_2_ lateral 2D heterostructures was established when the sulfur, molybdenum trioxide and tungsten precursors are used during CVD process (Fig. 5a, b) [89]. The developed 2D films had sharp atomic hetero-interfaces where the atomic plane shared the same crystalline orientation with armchair and zigzag orientation (Fig. 5c). The final morphology of the film was a triangular core–shell shape with MoS_2_ inside and WS_2_ outside (Fig. 5d). Raman characterization studies confirmed the fabrication of both vertical and lateral 2D films (Fig. 5f). Development *p-n* heterojunction between 2D WS_2_ and MoS_2_ film has intensified the photovoltaic effect of 2D hetero-structured films. Hence strong photoluminescence phenomenon was detected (Fig. 5e, g) [89]. A fairly sharp interface with the same crystalline orientation and lattice structure similar to WS_2_ and MoS_2_ 2D films was characterized. Furthermore, in another report CVD was employed to develop the lateral 1H MoS_2_/1T′ MoTe_2_ hetero-interfaces, where the two components had relatively large lattice mismatch [90]. There are several other cases, which demonstrated the development of heterointerfaces between 2D TMDC films with large lattice mismatch including 2D WSe_2_/MoSe_2_ [91] and MoS_2_/WS_2_ [92] confirming the capability of CVD method for deposition of semiconductor/semiconductor lateral 2D hetero-structures. Therefore, it can be concluded that the modern CVD technique has proven its versatile capabilities for successful deposition of 2D materials on organic and polymeric flexible substrates, insulators and graphene substrates. Table 1 summarizes the latest achievements of 2D TMDCs and their heterostructures fabricated by CVD technique on the wafer scale [93].

**Table 1:** Growth of 2D heterostructured films via CVD. Reprinted with permission from [93]

Type	Materials/substrate	Methods	Specifications
Vertical heterostructures	MoS_2_/WS_2_	one-step CVD: 10 mg of W + 100 mg of Te, 25 mg of MoO_3_, 500 mg of S; Si/SiO_2_ (285 nm); 100 cm^3^ Ar; 850 °C, 15 min; atmospheric	triangular MoS_2_ and WS_2_; type II band alignment
Vertical heterostructures	MoS_2_/graphene	graphene: Au foil, AP, CH_4_ 1.5 cm^3^, H_2_ 30 cm^3^, Ar 200 cm^3^, 970 °C MoS_2_: MoO_3_(530 °C), S (102 °C), substrate (680 °C), Ar/H_2_ (50/5 cm^3^)	monolayer graphene film at bottom; monolayer layer MoS_2_ at top; weak n-doping level
Lateral heterostructures	MoS_2_/h-BN	Ni−Ga/Mo foil substrate, NH_3_− H_3_ (110−130 °C), Ar/H_2_ 75 cm^3^, 30 min, 1000 °C; 4 cm^3^ H_2_S precursor, 680 °C, 25 min, 10 sccm Ar	with whole sizes up to 200 μm^2^
Lateral heterostructures	MoS_2_/WS_2_	one-step CVD: 10 mg of W + 100 mg of Te, 25 mg of MoO_3_, 500 mg of S; Si/SiO_2_ (285 nm); 100 cm^3^ Ar; 650 °C, 15 min; atmospheric	W_S_2 − MoS_2_ interface roughness is four unit cells with a width of 15 nm
Lateral heterostructures	MoS_2_/WSe_2_	step 1: 0.6 g ofWO_3_ 260 °C (Se), Ar/H_2_ (90/6 cm^3^) 20 Torr, 925 °C, 15 min; step 2: 0.6 g ofMoO_3_ 190 °C(Se), Ar (70 cm^3^) 40 Torr, 755 °C, 15 min	junction depletion width is ∼320 nm, type II band alignment
Lateral heterostructures	MoS_2_/MoSe_2_	0.7 g of MoO_3_, 0.4 g of S, 0.6 g of Se, 750 °C for sulfurization, 700 °C with 5 cm^3^ H_2_ for selenization, 15 min	triangular geometry thickness of 0.8 nm interface transition in scale of ∼40 nm
Lateral heterostructures	graphene/BN	graphene: Cu foil, APCVD, 1050 °C, Ar/H_2_ (930/60 cm^3^) CH_4_ 20 cm^3^ hydrogen etch graphene BN: NH_3_−BH_3_, 120 °C, 10−30 min	zigzag-oriented boundaries; sharp interface boundary with width of 0.5 nm

In general, the stability of the CVD fabricated 2D nanomaterials and heterostructures is quite high even though the information about the long-term stability of such nanostructures is rather limited on most of the published works. The long-term performance and stability of the CVD-grown TMDC-based electronic and optoelectronic instruments are greatly affected by the interfaces, defects and grain boundaries in the nanomaterials considering that they serve as carrier scattering centres. This in turn decreases the carrier mobility of the devices. Therefore, the preparation of 2D compound materials with clean surfaces, high quality and wafer-scale domain size should be a desired goal for future nano- and opto-electronics. It should also be stressed that a large number of other 2D compound materials have not yet been well studied or have not been successfully grown, and consequently, more exciting discoveries are waiting to be found.

### ALD of 2D Nanostructrures

ALD as a modification of CVD can implement various strategies toward thickness-controlled fabrication 2D semiconductors and sandwiched heterostructures. Although ALD was initially developed about 45 years ago, only at the beginning of the twenty-first century it getting more popularity owing to great advantages and development various precursors [94]. During the last decade, it has been used for fabrication of wide types of materials, including oxides, sulfides, selenides, tellurides, nitrides and metals [95]. A key requirement is that suitable molecular precursors must undergo self-limiting reactions, since the primary distinction between the ALD and other vapor deposition methods is the fact that a self-saturating surface monolayer is formed after every precursor exposure. A complete description of the latest developments in ALD chemistry can be found in recent reviews [94–96]. Thus, so far ALD has been implemented in a diverse number of fields including microelectronics [97], catalysis [98], photovoltaics [99] and sensors [100]. Unfortunately, the ALD implementation toward the synthesis of 2D semiconductors and 2D sandwich nanostructures is rather limited.

**Fig. 6:**
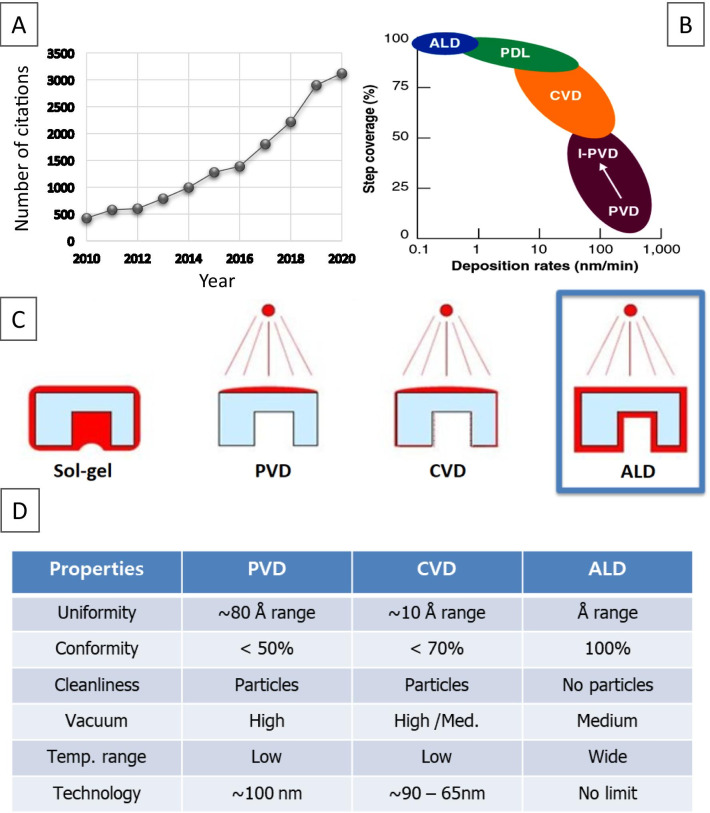
Number of annual citations on “Atomic layer deposition” topic **a** of published manuscripts. Data are according to Web of Knowledge™ Database. Comparison of the deposition rate vs step coverage (**b**) and coverage areas produced by different deposition techniques (**c**). Comparative analysis of properties of SE fabricated by PVD, CVD, and ALD, respectively (**d**). Reproduced with permission from [94]

As deposition technique, ALD consists of a series of self-limiting, surface-saturated reactions to form thin conformal films at a controllable rate. Despite all obvious ALD advantages, it has to be admitted that this emerging technology has not yet been fully exploited for fabrication of different 2D nanostructures. Apparently, the lack of reliable recipes and precursors, specific ALD temperature window for deposition are some of the valid reasons for that. On the other hand, it was recently reported that the ALD advantages are far superior to the existing capabilities of other deposition techniques [101]. Specifically, ALD is the only one technology, which enables fabrication of defects-free, conformal 2D nanofilms and their heterostructures on the wafer scale with precise, Ångstrom scale control of their thickness during deposition [102]. In this regard, the *state-of-the-art* nanoscale ALD interfacing and molecular engineering of 2D nanomaterials can open up completely new possibilities by providing ultra-thin channels for key doping, minimization of the density of interfacial impurities and optimization of capabilities of instruments. Furthermore, the ALD fabrication technique enables turning the design of 2D nanostructures toward heterojunctions and hetero-structures [103–105], and hybrid SE based on *inorganic–inorganic* [106], *organic–organic* and *inorganic–organic* nanomaterials [107]. Figure 6 illustrates growing interests to ALD as well as comparison of the deposition rates and properties for 2D nanostructures developed by various deposition techniques [94].

#### ALD of 2D Metal Chalcogenide Films

The layered TMDCs are composed of sandwiched structure where the metal layers covalently bonded with two individual surrounding chalcogen atoms, thus the layer-by-layer deposition mechanism in ALD technique is favourable for making layered 2D TMDC nanostructures. Among the different 2D TMDCs films including binary, ternary and doped chalcogenides, less than 20 type of them could be deposited via ALD method. The first MoS_2_ nanofilm was fabricated by ALD in 2015. Initially, the number of MoS_2_ layers was controlled by the tuning of parameters of ALD technique [106, 108–110]. Later, the ALD synthesis of TMDCs monolayer was reported [111–114]. The self-limiting layer synthesis (SLS) of MoS_2_ monolayer film was also facilitated based on the typical ALD half-reactions [115, 116]. Here, the some practical cases of ALD of stable 2D films of metal dichalcogenides are summarized in Table 2 [94].

**Table 2:** Review of the reported ALD deposition of ultra-thin 2D TMDC nanofilms. Reprinted with permission from [94]

Semiconductor materials	Precursors	ALD window	Materials structure
WS_2_	WF_6_/H_2_ plasma/H_2_S	300 °C–450 °C	Nanocrystalline
WS_2_	WF_6_/H_2_S + Si layer catalyst	300 °C–450 °C	Nanocrystalline
WS_2_	WC_l5_/H_2_S	390 °C	2H-phase
WS_2_	W(CO)_6_/H_2_S	175 °C–205 °C	Amorphous
WS_2_	WH_2_(iPrCp)_2_/O_2_ plasma	300 °C	Hexagonal
WSe_2_	WCl_6_/DESe	600 °C (5L), 700 °C (3L), 800 °C (1L)	Well-crystallized, hexagonal
Bi_2_S_3_	Bi(thd)_3_/H_2_O	300 °C	Orthorhombic
Bi_2_S_3_	Bi(thd)_3_/H_2_S	125 °C–300 °C	Polycrystalline orthorhombic
Bi_2_Te_3_	BiCl_3_/(Et_3_Si)_2_Te	160 °C–250 °C	Crystalline rhombohedral
Bi_2_Te_3_	Bi(NMe_2_)_3_/(Et_3_Si)_2_Te	70 °C, 120 °C	Polycrystalline rhombohedral
MoS_2_	MoCl_5_/H_2_S	300 °C	Well-crystallized, hexagonal
MoS_2_	MoCl_5_/H_2_S	330 °C–450 °C	Well-crystallized, hexagonal
MoS_2_	MoCl_5_/H_2_S	450 °C	Well-crystallized, hexagonal
MoS_2_	MoCl_5_/H_2_S	375 °C, 475 °C	Small crystallites
MoS_2_	Mo(NMe_2_)_4_/HS(CH_2_)_2_SH	50 °C	In H_2_: Nanocrystalline
MoS_2_	Mo(CO)_6_/H_2_S plasma	175 °C–225 °C	Polycrystalline 2H-MoS_2_
MoS_2_	(NtBu)_2_(NMe_2_)_2_Mo/O_3_	300 °C	2H-phase
MoS_2_	Mo(CO)_6_/O_2_ plasma	200 °C	Polycrystalline
MoS_2_	(NtBu)_2_(NMe_2_)_2_Mo/O_2_ plasma	150 °C	Function of the process
FeS*x*	Fe(amd)_2_/H_2_S	80 °C–200 °C	Well-crystallized
GaS	Ga_2_(NMe_2_)_6_/H_2_S	125 °C–225 °C	Amorphous
GeS	N^2^,N_3_-di-tert-butylbutane-_2,3_- diamine Ge (II)/H_2_S	50 °C–75 °C	Amorphous
InSe	InCl_3_/H_2_Se (8% in Ar)	310 °C–380 °C	Hexagonal (*γ*-InSe)
In_2_Se_3_	InCl_3_/(Et_3_Si)_2_Se	295 °C	Crystalline
γ-MnS	Mn(EtCp)_2_/H2S	100 °C–225 °C	T < 150 °C: (γ-MnS) *T* > 150 °C: (*α*-MnS)/*γ*-MnS
Sb_2_Se_3_	SbCl_3_/H_2_Se	270 °C–320 °C	Orthorhombic
Sb_2_Te_3_	SbCl_3_/(Et_3_Si)_2_Te	60 °C–140 °C	Crystalline rhombohedral
NiS	Ni(thd)_2_/H_2_S	175 °C–350 °C	Polycrystalline *β*-NiS
TiS_x_	TiCl_4_/H_2_S	400 °C–500 °C	Hexagonal on soda Amorphous on Rh
SnS	Sn(acac)_2_/H_2_S	80 °C–225 °C 80 °C–160 °C 390 °C	Long pulse: cubic Short pulse: orthorhombic *T* ≥ 300 °C: Orthorhombic
Bi_2_Te_3_/Sb_2_Te_3_	BiCl_3_/(Me_3_Si)_2_Te & SbCl_3_/(Me_3_Si)_2_Te	165 °C–170 °C	Polycrystalline,
InSe/Sb_2_Se_3_	InCl_3_/H_2_Se &SbCl_3_/H_2_Se	310 °C	–
WS_2_/SnS	WCl_5_/H_2_S& Sn(acac)_2_/H_2_S	300 °C	Hexagonal/ Orthorhombic

The first investigation of 2D MoS_2_ deposition was based on the sulfurization of ALD deposited MoO_*x*_ few-layered film. The formation of S–Mo-S arrangements of atoms along the *z*-axis was achieved after 10 min interaction of MoO_*x*_ film with S gas at 600 °C [117]. The morphology, uniformity, crystallinity, nucleation and the quality of ALD-deposited film have direct impact on the final properties and characteristics of the MoS_2_ film [117–121]. In this example, the parameters of multistep annealing process should be controlled precisely to facilitate the formation of MoS_2_ films with sequential reduction of Mo and then incorporation of S atoms. In addition, the multistep reactions allowed the coverage control and phase transformation from crystalline MoS_2_ to the 2H-phase at the higher post-treatment temperatures. The first wafer-scale CVD of MoS_2_ on the 2-inch sapphire substrate was reported in 2014, when MoCl_5_ and H_2_S were employed as the precursors in the ALD system at 300 °C [121]. It was found that 10 sequential ALD cycles are required to establish the uniform growth of the MoS_2_ film and prevent the islands growth. The following post-annealing of ALD MoS_2_ film at 800 °C improved the crystallinity of MoS_2_ at the expense of losing the thickness uniformity of MoS_2_ on the wafer substrates. Thus, higher number of ALD cycles were done (20 cycles) to compensate the lack of uniformity due to the high-temperature post-annealing process. Considering the ALD window, it was found that the ALD process within 350 to 450 °C permits higher quality of sulfide layers with the growth rate of 0.87 Å/cycles [122]. It was also demonstrated that the following annealing of as-deposited MoS_2_ film in H_2_S or sulfur gas can improve the stoichiometry of 2D MoS_*x*_ film [123].

The deposition of 2D MoS_2_ films via ALD technique can also be achieved by usage of other precursors. Other main precursors for deposition of MoS_2_ 2D films were Mo(CO)_6_, H_2_S and dimethyl disulfide compounds. The ALD window suitable for these precursors is in the range of 155–170 °C. By monitoring the mass gain during the ALD cycles, it was revealed that multiple pulses of Mo source are required to reach the saturation, while a single H_2_S injection is sufficient to saturate the reaction and achieve the growth rate of 2.5 Å/cycle in the optimized condition [124]. The plasma-enhanced atomic layer deposition (PE-ALD) can also be successfully employed to deposit ultra-thin *h*-MoS_2_ film on the different substrates. The growth rate of 0.5 Å/cycle was observed at the ALD window of (175–200 °C) for deposition of 2D MoS_2_ films. Lower ALD window (60–120 °C) caused the deposition of amorphous 2D MoS_2_ films. Particularly, the ALD at 100 °C allowed the formation of continuous layer of amorphous MoS_2_ on the Si wafer. To improve the crystallinity of as-deposited ALD film, the later rapid thermal annealing (RTA) treatment at 900 °C led to the formation of crystalline hexagonal layer of MoS_2_ [125, 126]. Another recipe for deposition of 2D MoS_2_ film is Tetrakis (dimethylamido) molybdenum (Mo(NMe_2_)_4_), which interacts with either H_2_S or alternatively 1, 2 ethanedithiol (HS(CH_2_)_2_SH) to deposit 2D MoS_2_ films. In this recipe, the ALD of MoS_2_ film taken place at the lower temperature (*T* < 120 °C) since the Mo(NMe_2_)_4_ decomposes at temperature higher than 120 °C. The as-deposited 2D film was amorphous in this technique. Thus, the subsequent annealing was employed to crystalize the 2D MoS_2_ films. The mass spectroscopy analysis revealed that the mechanism of ALD deposition of MoS_2_ film is based on the removal of the remaining NMe_2_ ligands from the substrate surface after interaction of sulfur gas with Mo precursor. It was roved that it is possible to deposited 2D films on polymeric substrates by using lower ALD temperature.

2D WS_2_ is another example of the layered dichalcogenide ALD deposited at 300 °C by using WF_6_ and H_2_S as precursors [127]. A Zn-based catalyst is required to initiate the deposition of WS_2_ film via ALD technique. Diethylzinc (DEZ) and H_2_S were employed to assist the rapid nucleation of WS_2_ film over a wide range of substrates [128]. Zn acts as reducing agent for WF_6_ favouring the adsorption of WF_6_ on the surface of substrates. The initial growth rate of 1.0 Å/cycle was recorded, which then was reduced after 50 ALD cycles of WS_2_ deposition. It was found that the deposition of a monolayer ZnS film is also facilitated the growth of WS_2_ film with crystalline structure [129, 130]

WSe_2_ is another TMDC 2D film which was fabricated by ALD [124]. In this report, a self-limiting approach for WCl_6_ and diethyl selenide (DESe) at the higher ALD temperature of 600–800 °C was employed and the dependence of film thickness on the ALD processing temperature was observed [131]. Five, three and one crystalized layers of WSe_2_ were deposited on the Si/SiO_2_ wafers (8 cm^2^) at 600 °C, 700 °C and 800 °C, respectively. After 100 ALD cycles, the growth of WSe_2_ was stopped due to growth saturation. The deposited WSe_2_ film with few layers and monolayer was crystallined with honeycomb structure. The same film was employed as a *p*-type semiconductor in the FET and showed the capability of ALD deposited WSe_2_ film for application in electronic devices. In another parallel attempt, the thermal ALD of continues WSe_2_ film on the Si/SiO_2_ substrate was successfully carried out at 390 °C [132]. WCl_5_ and H_2_Se were employed to deposit a highly uniform film with well-crystalline structure. The quality and crystallinity of the ALD WSe_2_ film were fairly compatible to the mechanically exfoliated and CVD WSe_2_ films [132].

SnS_x_ 2D family is the other types of 2D materials which was extensively studied during last few years. The first deposited SnS film was reported by using tin (II) 2,4-pentanedionate (Sn(acac)_2_) precursor as the Sn source and H_2_S as the sulfur source, respectively, where the growth rate of 0.23 Å/cycle was achieved in the ALD window from 125 to 225 °C. The other precursors for ALD of SnS film are bis(*N*,*N*’-diisopropylacetamidinato) tin (II) (Sn(amd)_2_) and N_2_,N_3_-di-tert-butylbutane-2,3-diamine tin (II) which react with H_2_S gas during ALD [133]. This recipe enabled the deposition of crystalline SnS film at the ALD temperature of less than 200 °C.

2D SnS_2_ and SnS_x_ films are deposited via employment of precursors containing Sn atoms [134]. The tetrakis (dimethylamino) tin (TDMASn) [135, 136] and tin acetate (Sn(OAc)_4_) [137] as the Sn (IV) metal precursors were used for ALD of SnS_2_ films. Polycrystalline hexagonal SnS_2_ films were deposited with the growth rate of 0.64–0.8 Å/cycle [134] at the temperature range of 140–150 °C. The growth of ALD temperature above 160 °C resulted in the development of thin mono-sulfide films. However, the as-deposited film always contains a combination of both mixed SnS and SnS_2_ compounds. Crystallinity of the film can still be improved by the following post-treatment annealing in H_2_S gas at 300 °C. As an example, the formation of a single phased orthorhombic 2D SnS_2_ films with interlayer spacing of 0.6 nm was reported after the post-annealing treatment at 300 °C in H_2_ atmosphere. Another precursor for ALD deposition of SnS_2_ film is Sn(OAc)_4_ where the ALD reaction in the ALD temperature of 150 °C resulted in the growth rate of 0.17 Å/cycle for the amorphous stoichiometric SnS_2_ film. Generally, in ALD deposition of few-layered SnS_x_ films with Sn precursors and H_2_S gas, the chemical composition, structure and the stoichiometry of SnS_x_ ALD structures can be modulated depending on the deposition conditions and the post-ALD treatments [137].

**Fig. 7:**
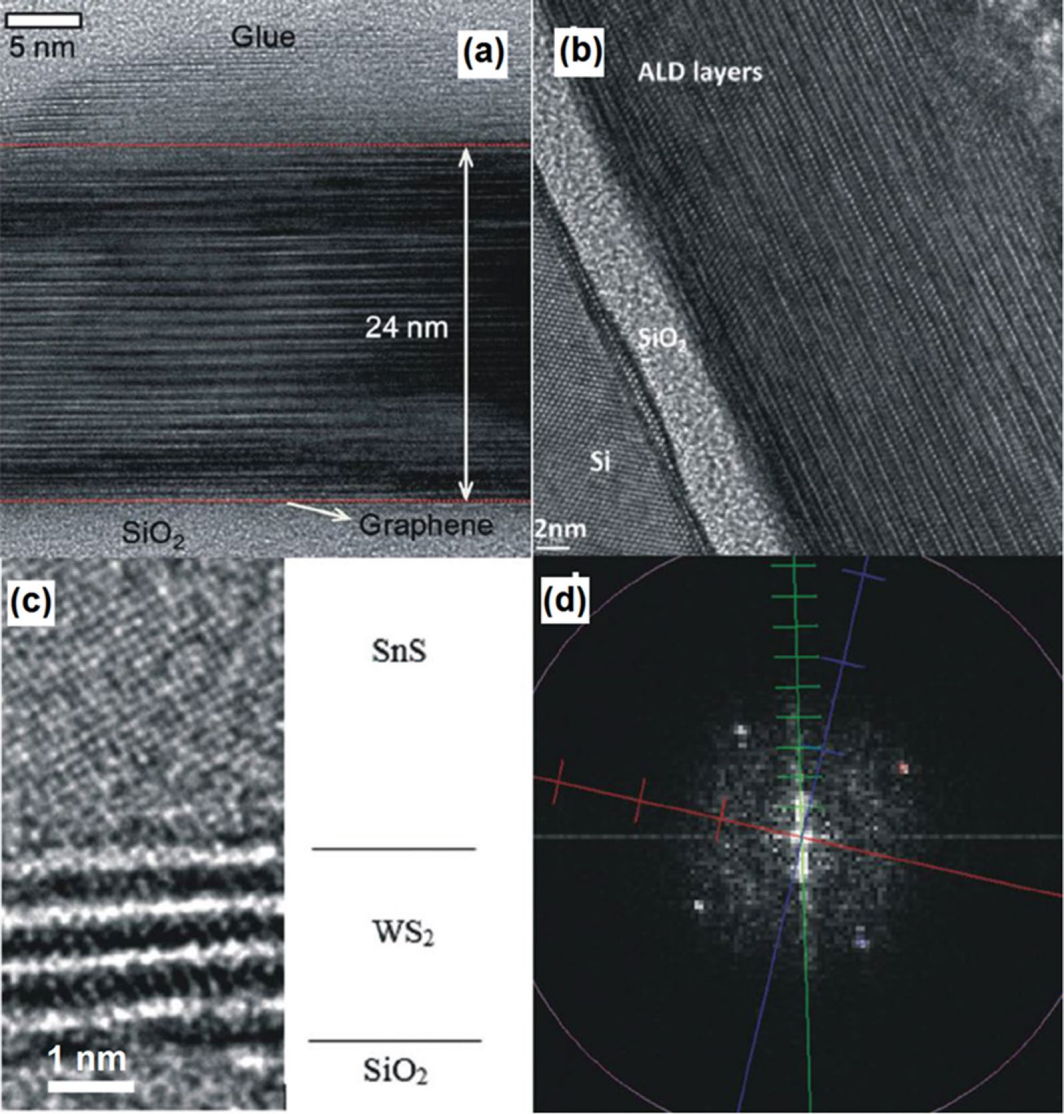
**a** Cross-sectional image from the interface between ALD grown Sb_2_Te_3_ on graphene/SiO_2_ substrate. **b** The cross-sectional image of heterostructured Sb_2_Te_3_/Bi_2_Te_3_ stacked layers. **c** The HRTEM image of SnS/WS_2_ heterointerfaces and **d** the corresponding fast Fourier transform (FFT) image. Reproduced with permission from [138]

One of the main ALD capabilities is the wafer-scale deposition of 2D hetero-structured TMDCs, which opens up further opportunity for fabrication of vdW hetero-structures. The technological challenges for deposition of 2D hetero-structured films are the main concerns for their deposition. Basal plans of the most of 2D materials are inert with the lack of dangling band, thus the nucleation of 2nd family of 2D films over the first layer is considerably challenging. Therefore, it is necessary to enable the initial adsorption of precursors on the surface of 2D film and then facilitate the growth of second 2D film over substrate. One of such examples is the ALD deposition of Sb_2_Te_3_ film on the surface of graphene by using SbCl_3_ and (Me_3_Si)_2_Te as precursors [138]. In this ALD process, (Me_3_Si)_2_Te was initially physically adsorbed on the graphene. The successful low deposition temperature of 70 °C for ~24.0-nm-thick crystalline Sb_2_Te_3_ layer was finally achieved with the crystalline plane parallel to the graphene substrates. A sharp hetero-interface was formed between the graphene and Sb_2_Te_3_ film as depicted on the high resolution transmission electron microscope (HRTEM) image in Fig. 7a [138].

In another attempt, 2D hetero-structured Sb_2_Te_3_/Bi_2_Te_3_ metal chalcogenides films were deposited by the ALD process on Si/SiO_2_ wafer. Here, the bismuth telluride was selected to be deposited over the silicon wafer by the ALD technique at 165–170 °C, since it covers the Si better than Sb_2_Te_3_. Considering the low ALD temperature of Bi_2_Te_3_ (at 65–70 °C), the employment of initial long SbCl_3_ pulse in the ALD chamber was taken as a capable strategy to saturate the surface of Bi_2_Te_3_ and complete the surface reactions for the next deposition step [139]. This approach also facilitated the disposition of three stacking layers of Bi_2_Te_3_/Sb_2_Te_3_ with a high level of crystallinity. Another example is the growth of multiple WS_2_/SnS layered semiconductor hetero-junctions on the Si/SiO_2_ substrates by alternating ALD of WS_2_ and SnS at the ALD temperature of 390 °C [140]. The stacked 2D WS_2_/SnS has the 15° orientation angle between their *c* axis originated from the difference in crystalline structures and the lattice mismatch between WS_2_ and SnS (Fig. 7b, c) [133]. This misaligned crystalline characteristics of WS_2_/SnS hetero-interfaces is the origin of noticeable decreased of the holes mobility in SnS film [140]. Generally, the ALD techniques are recently undergoing consistent progress to enable the deposition of higher numbers of 2D TMDC films. However, there are still countless research opportunities to develop suitable ALD recipes for deposition of 2D TMDC films over the different substrates and also to develop nanoelectronic instruments based on 2D TMDCs.

#### ALD of Ultrathin Oxide Films

The few-nanometer-thick metal oxide films can be considered as 2D nanostructures when they can be assigned to few layers of 2D films. In this context, the characterization and exploration of 2D oxides properties for the practical applications are considered as valuable technical knowledge along with the progressive advancement in the device miniaturization. Furthermore, modern technologies use the advantages of ultra-thin metal oxide nanostructures as the main components of *state-of-the-art* nanodevices. Consequently, promising applications for the ultra-thin 2D semiconductors and insulators are expected, such as solid oxide fuel cells, catalyst films, corrosion protection layers, chemical sensors, spintronic devices, and UV and visible light sensors. Metal oxide semiconductor field-effect transistors (MOSFET) and the other novel nanoelectronic instruments are fundamentally dependent on the ultra-thin uniform films of metal oxides. Complementary, metal oxide semiconductor (CMOS) sensors are technically developed based on the deposition of ultra-thin metal oxide films which opened up a quite number of the different applications from image and light sensors to FET transistors in low-energy semiconductor instruments. The solar energy cells, plasmonic devices, data storage applications, bio-compatibility and bio-sensing features, supercapacitance properties, and electrochemical sensing are among the recently announced and discovered applications of the 2D oxide semiconductors. Thus ALD deposition of 2D metal oxide semiconductor thin films is considered as advanced fabrication technology. It is also an extremely difficult target to achieve considering the fact that the structural stability and molecular integrity of 2D films can be deteriorated easily. Accordingly, one of the main challenges is the versatile and conformal deposition of these 2D oxide nanostructures on the convenient substrates [94]. The following section is specifically focused on the ALD of metal oxide materials, and particular attention is given to molybdenum, titanium, aluminum, and tungsten oxide 2D films.

##### MoO_3_

**Fig. 8:**
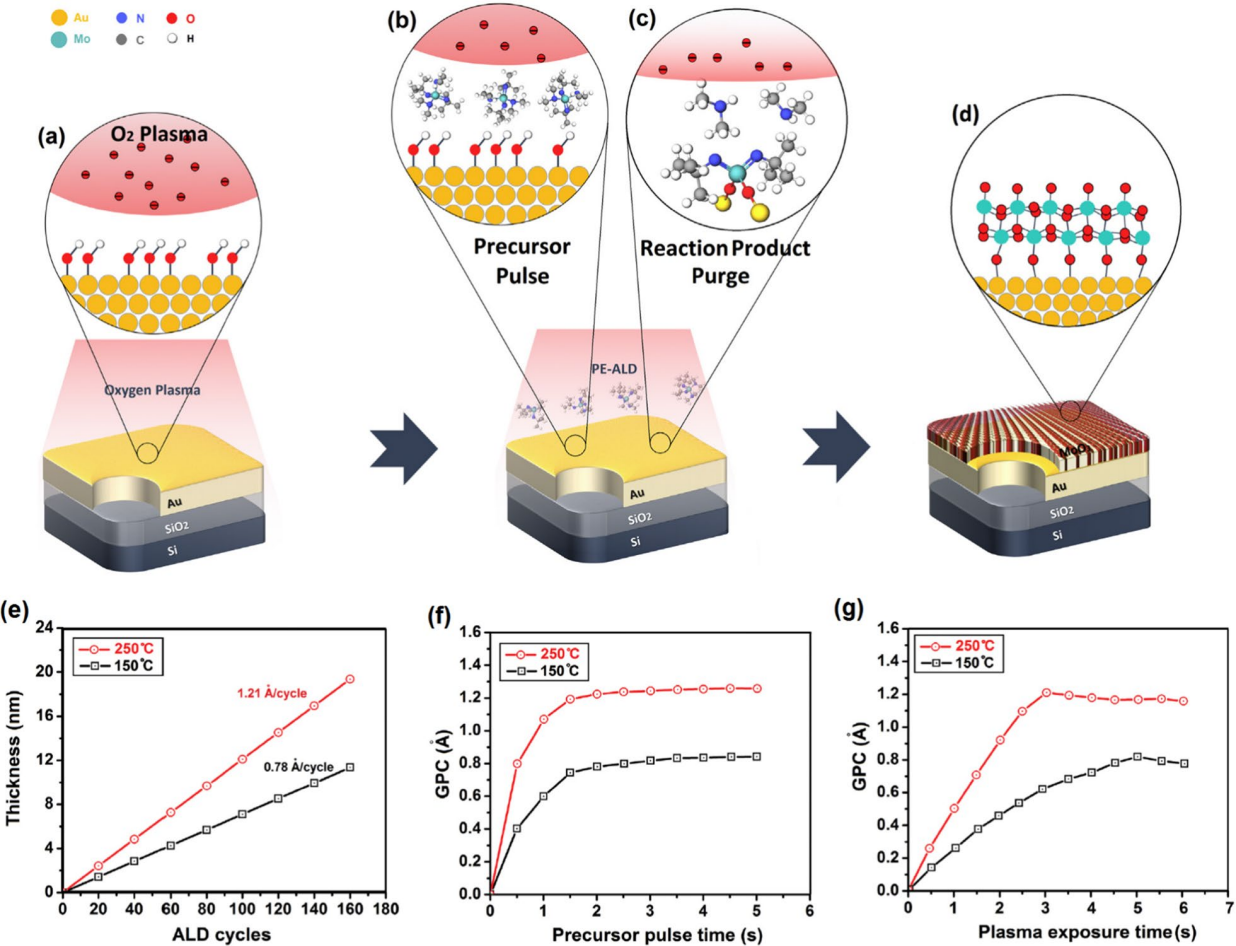
**a** Graphical scheme of deposition of 2D MoO_3_ nanofilms by PE-ALD. **b** The injection of precursor into ALD chamber and **c** the reaction between precursor and Au surface (**d**) and the complete deposition of a monolayer film over Au substrate. **e** The thickness of PE-ALD MoO_3_ film over Au substrate vs. the ALD cycle number at based on the usage of (NtBu)_2_(NMe_2_)_2_Mo precursor and O_2_ plasma at 150 °C and 250 °C, with the precursor dosing time of 2 s and plasma exposure time of 5 s. **f** The saturation curve of (NtBu)_2_(NMe_2_)_2_Mo precursor. The GPC is demonstrated as the function of precursor dosing time. **g** The saturation curve for O_2_ plasma showing the GPC as the function of plasma exposure time. Reproduced with permission from [141]

Distinguishable properties of nanostructured molybdenum trioxide (MoO_3_) made it an excellent candidate for catalytic, electrical and optical applications. There are also several promising functionalities originated from the heterointerface between the MoO_3_ semiconductor and their metallic substrates. The bis(tert-butylimido) bis(dimethylamido) molybdenum ((tBuN)_2_(NMe_2_)_2_Mo) is one of the precursors for MoO_3_ deposition, since it provides good volatility and thermal stability [141]. The PEALD window for molybdenum oxide with O_2_ plasma and ((tBuN)_2_(NMe_2_)_2_Mo) precursor is in the range of 50–350 °C. The as-deposited films fabricated at the temperatures below 250 °C are usually amorphous, thus the post-deposition annealing process is needed to experience a transition from the amorphous to crystalline state. While the as-deposited films are sub-stoichiometric, the stoichiometry can be adjusted by the modulation of plasma parameters. The other precursors for ALD of ultra-thin MoO_3_ film is Mo(CO)_6_ which is mostly employed via O_2_ plasma to facilitate the low-temperature PE-ALD of MoO_3_ films in the narrow ALD window of 152–172 °C [141]. The resulted MoO_3_ film was deposited with growth rate of 0.75 Å/cycle. The as-deposited film was found amorphous with the oxygen deficiency at the heterointerface between substrate and MoO_3_ film. To achieve the crystalline structure, the following post-annealing at temperature above 500–600 °C is required. In another strategy, the ozone plasma was used on the surface of ALD MoO_3_ film deposited on 300 mm Si/SiO_2_ wafer [141]. Mo film was fabricated by using Si_2_H_6_ and MoF_6_ precursors at 200 °C. The wafer-scale deposition of ultra-thin MoO_3_ film over 4-inch Au on the Si/SiO_2_ wafer was reported, where C_12_H_30_N_4_Mo as molybdenum precursor and O_2_ plasma as oxygen source were used at two ALD temperatures of 150 and 250 °C, respectively. The process includes the pre-exposure of bare Au surface by O_2_ plasma which assists the formation of OH bonding on the Au surface. The following chemisorption of C_12_H_30_N_4_Mo molecules by the active sites on the Au and interaction with OH group on the surface of Au film led to the ligand exchange. The following O_2_ plasma completed the oxidation process and finalized the growth of ultra-thin monolayer MoO_3_ film (Fig. 8). The development of Mo–O-Mo bonding is expected after completion of oxidation process. Finally, 4.6-nm-thick MoO_3_ film was deposited over the wafer substrate. In this technique, the growth rate from 0.78 Å/cycle to 1.21 Å/cycle was measured when the ALD temperature increased from 150 to 250 °C [141].

##### WO_3_

**Fig. 9:**
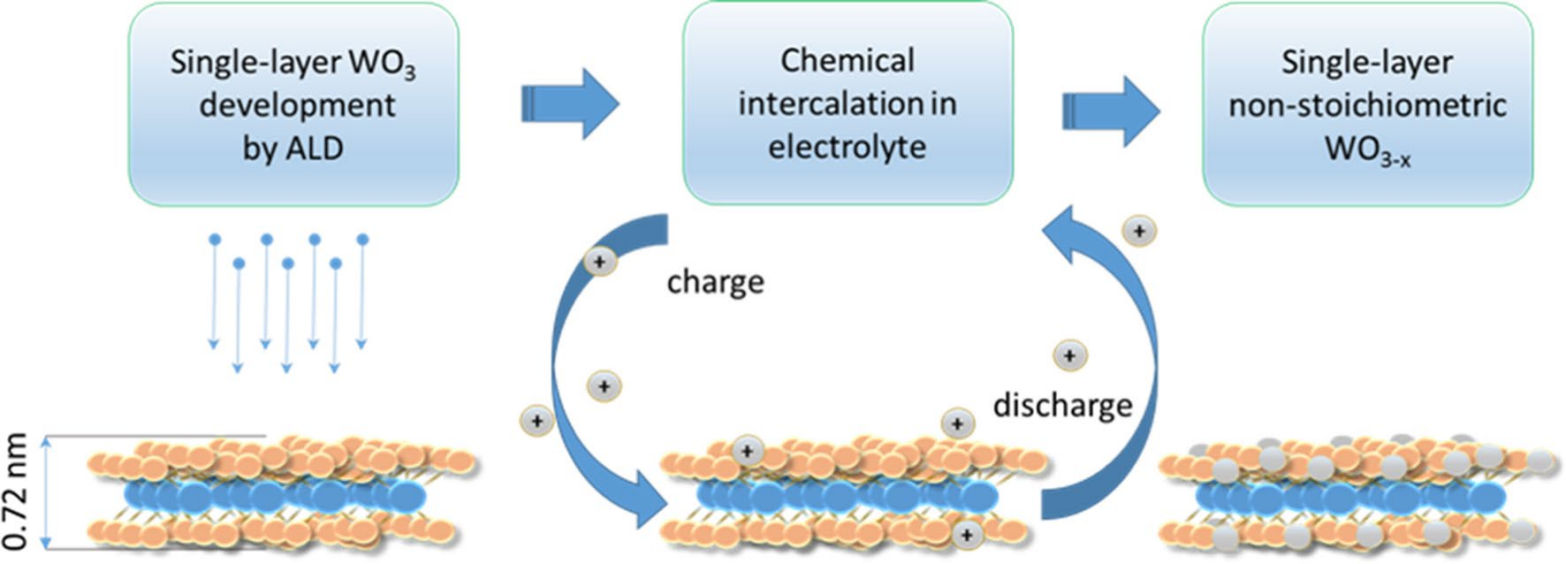
Enhancement of non-stoichiometry in monolayer WO_3_ film developed by ALD via intercalation/de-intercalation process

An uniform ultra-thin film of tungsten oxide (WO_3_) is one of the most interesting transition metal oxide semiconductors, which was successfully employed for significant number of optical, energy and environmental applications. Up to know, several precursors are introduced for successful deposition of WO_x_ film over the different substrates. One of the well-recognized recipes for ALD of WO_3_ thin films is tungsten hexacarbonyl W(CO)_6_ where a narrow ALD window of 195 and 205 °C with a GPC of 0.2 Å/cycle were noted when the ozone was used as the oxygen source in the ALD process [142, 142]. Due to low temperature ALD, the thin film is amorphous, and consequently, it needed the post-annealing process to improve the level of crystallinity of WO_3_ film [143]. Another ALD recipe was recently developed for the wafer-scale deposition of atomically thin WO_3_ film. In this recipe The bis(ter-butylimido) bis(dimethylamino) tungsten(VI) (^t^BuN)_2_W(NMe_2_)_2_ as tungsten precursor and H_2_O as oxygen source were also used [144]. This W source presented a low vapor pressure suitable for ALD technique. It was frequently observed that the post annealing can improve the crystallinity of ALD film in the expense of losing the uniformity and integrity of 2D nanostructures. Here was also found that the post annealing process improved the crystallinity, stoichiometry and electrical properties of 2D WO_3_ films. From the structural point of view, when the post annealing was performed at the low temperatures (*T*<300 °C), the 2D ALD film kept its mechanical stability on the substrate. However, by the increase of the post annealing temperature to higher values (*T*>300 °C) the nucleation and the growth of granular WO_3_ nanostructures were observed. Further increase in annealing temperature or annealing time led to deterioration of integrity of 2D films over the substrate, and it finally resulted in the growth of coarse granular WO_3_ nanostructures [144]. Figure 9 shows approach to enhancement of non-stoichiometry in monolayer WO_3_ film developed by ALD via intercalation/de-intercalation process.

##### TiO_2_

Wafer-scale deposition of ultra-thin titanium dioxide (TiO_2_) film on the Si/SiO_2_ substrate was achieved by using the using tetrakis (dimethylamino) titanium (TDMAT) precursor and H_2_O as an oxidation agent [145]. The optimum ALD temperature of 250 °C was selected for ALD of monolayer TiO_2_ film on the wafer-scaled substrate. To achieve a complete coverage of monolayer TiO_2_, two individual ALD super-cycles were designed and implemented [145]. The first super-cycle consisted of 10 consecutive cycles of pulse/purge TDMAT to assure the reliable coverage of surface by precursors. Afterward, 10 pulse/purge stages of H_2_O were performed to assure the completion of oxidation [145]. The XPS studies confirmed the changes of binding energies in both Si 2p and Ti 2p peaks after the deposition of 2D TiO_2_ could be elucidated by the development of Si–O–Ti bonds at the interface between native SiO_2_ and deposited 2D TiO_2_ film. The deposition of TiO_2_ on SiO_2_ substrate was accompanied by the decrease in binding energy of Si^4+^ which was related to this fact that the silicon was more electronegative than titanium. The evidence of oxygen bridge bonding was also demonstrated by characterization of vibrational mode of Ti–O–Ti bonds in the FTIR spectrum of monolayer TiO_2_ and also by investigation of XPS spectrum of monolayer film [145]. The bandgap of 3.37 eV was measured for this TiO_2_ film with atomic-scale thickness. The capability of ALD for deposition of ultra-thin films is of great importance to functional applications like microelectronics and catalysis where only few ALD cycles are desirable. Table 3 summarizes practical cases for ALD development of various 2D metal oxides reported to date.

**Table 3:** Review of the reported ALD deposition of ultra-thin 2D metal oxide films. Reprinted with permission from [94]

Semiconductor materials	Precursors	ALD window	Materials structure
WO_3_	W(CO)_6_/H_2_O_2_	180 °C–200 °C	Amorphous
WO_3_	W(CO)_6_/O_3_	195 °C–205 °C	Partially crystalline as-deposited Crystallinity enhanced after annealing
WO_3_	W(CO)_6_/H_2_O	150 °C–320 °C	Amorphous layer completely crystallizes into polycrystalline film under post-annealing
WO_3_	(tBuN)_2_ W(NMe_2_)_2_/ H_2_O	250 °C–350 °C	Crystalline
	(tBuN)_2_ W(NMe_2_)_2_/ H_2_O	300 °C–350 °C	Amorphous as-deposited
WO_3_	WO_2_(tBuamd)_2_/H_2_O	120 °C–270 °C	Crystallize as WO_3_ nanowires
WO_3_	WF_6_/H_2_O	30 °C–180 °C	Amorphous
W_2_O_3_	W_2_(NMe_2_)_6_/H_2_O	140 °C–200 °C	Amorphous
Al_2_O_3_	TMA/H_2_O	180 °C	Amorphous
AlO_x_	TMA/H_2_O	90 °C	Amorphous
TiO_2_	TiCl_4_/H_2_O or O_2_	30 °C–180 °C	Amorphous
TiO_2_	TDMAT/H_2_O	150 °C	Amorphous
TiO_2_	TDMAT/H_2_O	250 °C	As-deposited amorphous, Annealed crystalline: *T* > 280 °C Anatase T > 400 °C rutile
ZnO	DEZ/H_2_O	200 °C	Single-layer ZnO on graphene presents graphene-like structure instead of wurtzite structure
MoO_3_	(tBuN)_2_(NMe_2_)_2_Mo/O_2_-PEALD	50 °C–350 °C	Amorphous films at < 200 °C Polycrystalline at > 250 °C
MoO_3_	(tBuN)_2_(NMe_2_)_2_Mo/O_2_-PEALD	150 °C	2H-MoS_2_ after annealing at *T* > 900 °C
MoO_3_	(tBuN)_2_(NMe_2_)_2_Mo/O_2_-ALD	300 °C-	2H-MoS_2_ after annealing at *T* > 900 °C
MoO_3_	Mo(CO)_6_/O_3_	152 °C–175 °C	Amorphous as-deposited α- and β-MoO_3_ phases after annealing at 500 °C
MoO_3_	MoO_2_(R_2_amd)_2_ (R = Cy; iPr)/O_3_	150 °C–225 °C	Amorphous
MoO_3_	CoCp_2_, Co(thd)_2_ or Mo(CO)_6_/O_3_, H_2_O or (O_3_ + H_2_O)	167 °C	Amorphous as-deposited Crystallize into *β*-CoMoO_4_ under annealing

In regard to stability of the ALD fabricated 2D nanostructures and their heterointerfaces, its mainly depends on the surface chemistry of ALD of 2D materials, especially because the inertness of their basal plans is expected to inhibit their growth and thus formation of vdW heterostructures. Moreover, as each precursor has specific “*ALD window*” of deposition temperatures, *trials and errors* approach is required for optimization of the ALD fabrication temperature for each specific precursor taking into account the final thickness and porosity of deposited materials [98, 109]. Owing to the low deposition temperature, ALD of 2D oxides usually leads to fabrication of amorphous layers [32, 94]. Hence, when the crystalline phases of metal oxides are needed, post-annealing treatment is generally applied, improving the crystalline quality of the films as well as the interfaces with the substrate, which ultimately leads to the better stability of 2D nanomaterials over the time. Finally, although ALD appears to be a method of choice to fabricate oxide monolayers due to its precise control of the film thickness, only a handful of reports can be found in the literature [21, 32, 98, 141, 144, 145]. Therefore, the area of 2D metal oxide and their hetero-interfaces fabricated by ALD remains rather unexplored.

### Self-limiting 2D Surface Oxides of Liquid Metals

The library of novel 2D materials for advanced applications is currently limited by the conventional synthesis methods of 2D films. The mechanical exfoliation of 2D films from their host layered bulks is not technologically precise and attractive method since the sizes distribution and thickness of produced nanosheets are not adequately and accurately controlled [146]. While the high vacuum techniques are accuracy of the depositing films are considered as high-quality films, these methods are still expensive, time consuming, and restricted by availability of precursors [64]. On the other hand, it is always desirable to introduce new synthesis methods of 2D materials in order to obtain the advanced 2D nanostructures with superior properties. Thus, synthesis of novel 2D semiconductor materials with distinguished or improved characteristics still remains a fundamental challenge.

Room-temperature liquid metals are a group of metals and alloys with unique electron-rich metallic cores which let them to stay in the liquid states even at the room temperatures. The interface of liquid metals with surrounding environment is the origin of one of the most natural 2D films [147]. This self-limiting surface oxide films (skin) is one of the most perfect planar materials with atomic scale thickness [148]. These crystalline structures are the host of unique properties and have provided a novel platform for synthesis of high-quality thin-film 2D materials for advanced applications [149]. Here, the liquid metals have the role of host material for synthesis of 2D films. With respect to the atomic structure, elemental composition, fluidity and thermodynamic of liquid metals, and furthermore, by considering the nucleation and growth characteristics of the surface oxide films, countless parameters are engaged to determine the proprieties of extracted 2D surface oxide films [150]. The low melting monophasic metal alloys are composed of post transition metals (Ga, In, Sn, Pb, Al, and Bi) and the elements of group 12 (Zn, Cd, and Hg). These elements can be combined together to produce a group of liquid alloys with low melting temperature. A biphasic liquid metal (mixture of solid and liquid) can be formed by either deviation of alloy composition from eutectic point or via adding solute elements above the solubility limit into the liquid metal solvent [151]. Gallium (Ga) is one of the most famous liquid metals with room-temperature melting point and possessing both covalent and metallic bonds at the solid state. The strong structural anisotropy and weak atomic bonding between Ga dimmers is originated from the distance between Ga neighbour atoms 2.7–2.9 Å which is considered as a significant distance between the gallium dimers [152]. This weak atomic bonding results in the weak crystalline structure in gallium which can easily break up at the room temperatures [153]. The alloying of Ga with some of the other elements may lead to further decrease in melting temperature of liquid alloy. One of the famous examples is the Ga-In alloy, when the melting temperature decrease to 16 °C in eutectic point of In-Ga alloy with atomic concentration of 14.2% indium [153]. The interface between gallium alloy and surrounding atmosphere is composed of the atomic-scale-thick gallium oxide (Ga_2_O_3_). The liquid metal reacts with atmospheric oxygen even at very low oxygen pressure forming a self-limiting metal oxidation reaction [153]. This ultra-planar oxide films are among the most perfect naturally grown 2D materials [61]. This natural film prevents the liquid metal from the further oxidation. The ionic transfer through the natural surface oxide film is the controlling factor for the natural growth of surface oxide films of liquid metal and alloys. The formation of this oxide film leads to the highest reduction in the Gibbs free energy (Δ*G*_f_) [154]. In a liquid metal alloy, the surface oxide film is mostly composed of surface oxide films of one of elemental oxide films of alloy components. The most famous example is the surface of oxide film of galinstan alloy. Galinstan (EGa, In, Sn) is the eutectic alloy of gallium–indium–tin. However, the surface oxide of galinstan is composed of Ga_2_O_3_. Since the Δ*G*_f_ of Ga_2_O_3_ is lower than that of In_2_O_3_ and SnO_2_ in oxygen atmosphere, the surface oxide of galinstan alloy is mostly composed of Ga_2_O_3_ [155]. With the same synthesis strategy, it is possible to synthesize the mixed 2D oxide films similar indium–tin–oxide. To this aim, the molten In–Sn alloy should be used to extract 2D films from the surface of liquid In–Sn alloy [154].

Due to the nonpolar properties of liquid metals, the attraction force between the liquid metal and its natural surface oxide film is weak and localized. Thus, a weak mechanical force can simply delaminate the surface oxide films from the surface of liquid metals. The delamination of surface oxide film can be achieved via the mechanical separation and exfoliation of 2D films by applying mechanical exfoliation methods including sonication, touching of liquid metal with the appropriated selected substrate, mechanical rolling of the liquid metal and alloy over the smooth substrate and separation and extraction of 2D films through the density differences and gradients between the synthesized compounds. These methods were employed recently for delamination of several different types of the surface oxide films of liquid metals from their host alloy. The following section will review some of lately synthesized 2D surface oxide films of liquid metals and alloys.

#### *SnO*_*x*_

2D SnO_x_ film was successfully delaminated via vdW exfoliation of the liquid tin surface oxide film. Elemental tin (Sn) with its low melting point (231.9 °C) [156] and its high conductivity (9.17 × 10^6^ σ) is used as electrical connections in the electronic utensils. The anti-corrosion impact of tin oxide layer has been confirmed before. SnO_2_ naturally covers the surface of tin alloy under the atmospheric condition and prevents further oxidation of Sn [156]. It was found that the natural surface oxide film of liquid tin in the ambient atmosphere is composed of a mixture of tin oxides which is predominantly composed of SnO_2_, with some contributions of SnO, Sn_2_O_3_ and Sn_3_O_4_ [156]. The precise control of oxygen exposure to the surface of fresh molten tin is the main technical factor to determine the composition of surface oxide film of tin alloy. This is a highly important factor during the synthesis of the surface oxide film of molten tin, when we take into consideration the fact that the SnO_2_ is *n-*type semiconductor, and SnO is a *p*-type semiconductor with a variety of different electrical properties. The bipolar characteristics of SnO is another important properties of this material which made it a functional choice for application in electrical invertors [157]. Furthermore, the high stability and sensitivity of well-established SnO films turned them to the desired option for application in chemical-based FET sensors and catalyst films. The transparency is another characteristic of SnO films originated from the wide bandgap of this semiconductor [156, 157].

**Fig. 10:**
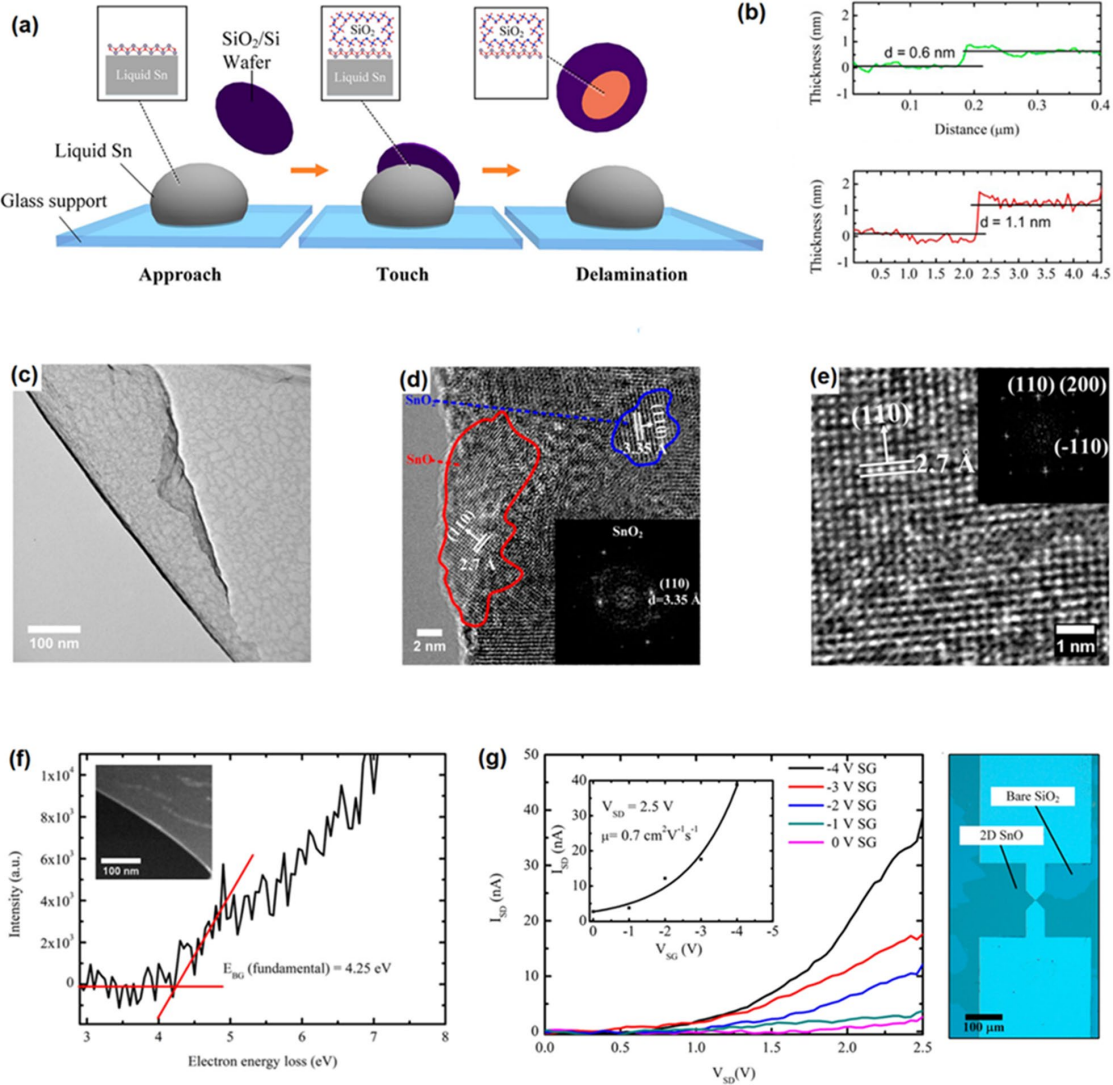
**a** Graphical representation of vdW exfoliation method. When a Si/SiO_2_ substrate is attached to the molten tin alloy, the created vdW force between the tin oxide and SiO_2_ surface help to delamination and following attachment of 2D oxide film to the SiO_2_ substrate. **b** The AFM image of the monolayer (0.6 nm) and bilayer (1.2 nm) tin oxide film. **c** The TEM image of tin oxide film developed in controlled atmosphere. **d** The HRTEM of tin oxide film developed in ambient atmosphere with mixed SnO and SnO_2_ structure. **e** The HRTEM image of SnO film developed in controlled atmosphere. **f** The bandgap of monolayer SnO film measured by electron energy loss spectroscopy. **g** The *I-V* characteristics of fabricated SnO FET device at various gate voltages. Reprinted with permission from [156]

To exfoliate2D SnO_x_ film, the fresh surface oxide film of molten tin was mechanically delaminated by the contact of surface of Si/SiO_2_ substrate with the surface of molten alloy inside of the glove box [158]. The scheme of process was graphically demonstrated in Fig. 10a [158]. The delaminated SnO_x_ film in ambient atmosphere had ~ 0.6 nm thickness [157] with crystalline structure. Considering the interlayer spacing of crystalline SnO (0.484 nm), 0.6-nm thick film can be considered as a monolayer SnO film and 1.1-nm-thick film is therefore a bilayer SnO 2D film (Fig. 10b). The 2D films synthesized in glovebox and under oxygen concentration of 10–100 ppm had ultra-fine surface characteristics. The measurement of interlayer spacing showed that the nanosheets formed under ambient atmosphere were composed of two distinct materials with interlayer spacing of 2.7 Å and the other 3.35 Å, which were respectively attributed to the crystal lattice of SnO and SnO_2_. The 2.7 Å lattice spacing was related to the (110) plane of SnO, while the 3.35 Å lattice spacing was attributed to the (110) plane of SnO_2_, respectively (Fig. 10c–e) [158]. The sample synthesized in the reductive atmospheres was mostly covered by SnO film. The bandgap measurement of SnO film showed the direct bandgap of 4.25 eV. This ultra-thin 2D film was employed to fabricate the SnO-based FET transistor. The *p*-type characteristics of SnO-based developed FET was confirmed with the mobility of 0.7 cm^2^V^−1^ s^−1^ (Fig. 10g) which was much higher than the similar FET devices based on the pulsed layer deposited bilayer SnO film with the mobility of 0.05 cm^2^V^−1^ s^−1^ [159].

#### *GaPO*_*4*_

**Fig. 11:**
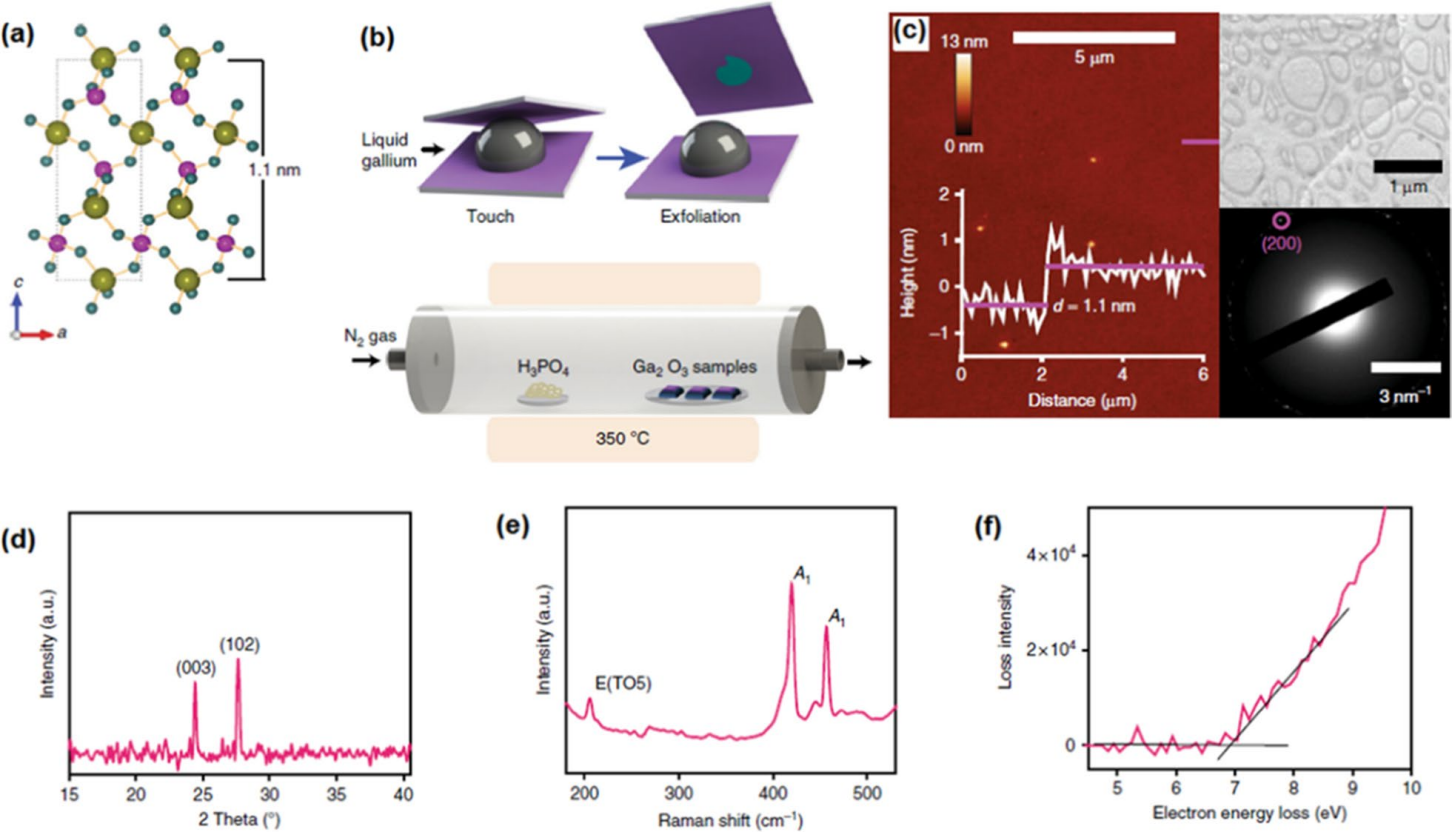
**a** The side view of GaPO_4_ demonstrating the out-of-plane structure of GaPO_4_. **b** For the secondary phosphatization treatment. **c** The AFM and HRTEM studies of 2D GaPO_4_ film. **d** The XRD, **e** Raman and **f** bandgap measurement of 2D GaPO_4_ film. Reprinted with permission from [160]

The high-temperature stable gallium phosphate (GaPO_4_) semiconductor (up to 930 °C) is one of the functional piezoelectric semiconductors with outstanding technical position compared its rival of α-quartz [61]. Gallium phosphate has trigonal structure with the cell parameters of *a* = 4.87 Å and *c* = 11.05 Å and *γ* = 120° (Fig. 11a) [160]. The piezoelectricity facilitates the mutual conversion of electrical energy or pulses to mechanical forces or oscillations. To develop miniature power and electric instruments and generators, it is highly required to synthesize 2D piezoelectric materials for novel nanoelectronic technology. In this device, the atomistic level vibration, bending and displacement can be monitored and then facilitate the harvesting of kinetic energies of oscillation of piezoelectric materials. The loss of centro-symmetry is the main structural specifications of 2D materials with piezoelectric properties [160]. Many types of 2D materials including, TMDCs with odd number of layers, transition metal oxides, aluminium nitride, GeS and SnSe_2_, *h*-BN and graphene are capable of showing the piezoelectric properties [161–163] at the low temperatures. Nowadays, it is highly required to synthesize new 2D materials with stable piezoelectric performance at high operational temperatures. This is very challenging issue since the performances of 2D piezoelectric materials are failed due to the structural changes at the high working temperatures. Another difficulty arises from the challenging deposition stage of homogeneous 2D piezoelectric films of functional instruments.

As a successful achievement, the 2D archetypal piezoelectric GaPO_4_ film was recently synthesized via processing of 2D surface oxide film of gallium. The archetypal piezoelectric GaPO_4_ is not naturally available in crystalline scarified structure [160], and thus the conventional mechanical exfoliation techniques are not capable of synthesis of 2D crystalline gallium phosphate films. In this method, natural surface oxide film of gallium was mechanically exfoliated from its liquid alloy via vdW delamination technique, see Fig. 11b. The secondary post-annealing treatment was employed inside a furnace for the secondary phosphatiation treatment [61]. The vapor gas containing phosphor chemically interacted with the surface of 2D Ga_2_O_3_ film by the heating of H_3_PO_4_ powders. The N_2_ gas had the role of carrier gas for transfer of vapor phosphor gas to the surface of 2D Ga_2_O_3_ film at 300–350 °C (Fig. 11b). The following AFM and HRTEM measurement of samples confirmed the synthesis of 1.1-nm-thick 2D film with dimensional characteristics similar to the trigonal GaPO_4_ in the *c* crystalline direction (Fig. 11c) [160]. The XRD studies and Raman characterization have confirmed that 2D GaPO_4_ film had crystalline structured with dominance of (003) and (102) planes of trigonal α-GaPO_4_. (Fig. 11d, e). A wide bandgap of 6.9 eV was measured for α-GaPO_4_ film (Fig. 11f). The experimental measurements reaffirmed the strong out-of-the-plane piezoelectric properties for the GaPO_3_ 2D nanostructures which was around 8.5 pm V^−1^ [160]. This considerable piezoelectric characteristics of 2D α-GaPO_4_ highlighted the capability of this synthesis method to develop novel 2D materials with distinguishable mechano-electric properties.

#### GaN

**Fig. 12:**
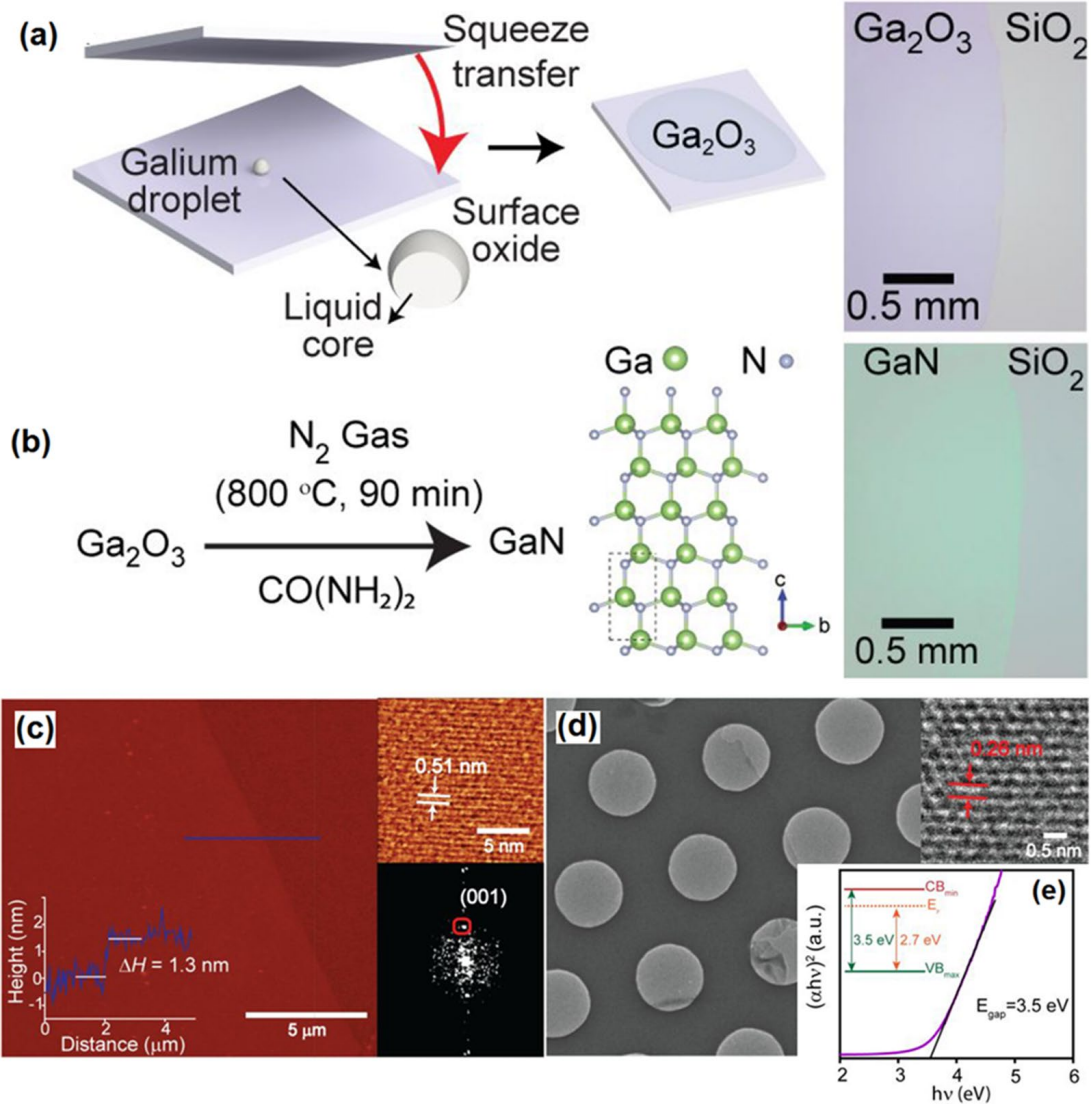
**a** Schematic illustration of squeezing of liquid metal galinstan between SiO_2_ substrate and **b** following post-ammonolysis treatment for phase transform of Ga_2_O_3_ to GaN film. **c** The High resolution AFM image of the surface of GaN film. **d** The HRTEM image of GaN film and **e** the band gap of measurement of GaN film. Reprinted with permission from [160]

The same post-processing approach was employed to synthesize GaN and InN 2D films from the surface oxide film of liquid gallium and indium [160]. The technique is based on squeeze printing of liquid gallium droplet between the Si/SiO_2_ parallel substrates (Fig. 12a). In contact with atmosphere, Ga liquid was instantaneously oxidized in atmospheric condition. The synthesis process resulted in the formation of 2D Ga_2_O_3_ films with thickness of 1.4 nm [157]. The following ammonolysis reaction at 800 °C was operated in the presence of urea. It was found that lower reaction temperatures did not facilitate the conversion of Ga_2_O_3_ into the wurtzite GaN films (Fig. 12b) [160]. The thickness of the film after ammonolysis process was found to be 1.3 nm which is corresponded to the to three wurtzite GaN unit cells (Fig. 12c). The lattice constant of 5.18 nm was measured for 2D GaN film. The TEM studies also confirmed the crystalline structure of GaN with interlayer spacing of 0.28 along the (001) crystalline plate (Fig. 12d) [160]. The X-ray photoelectron spectroscopy (XPS) affirmed the substantial amount of nitrogen was replaced by the oxygen atoms in GaN structure which is owing to the similar bond length of Ga-O and Ga-N. It was targeted to decrease the level of oxygen in 2D GaN films. However, the successful removal of oxygen from the 2D GaN films was not achieved even during the synthesis process at the high temperatures. A bandgap of *E*_g_ = 3.5 eV was measured for 2D GaN film (Fig. 12e) compared with that of Ga_2_O_3_ film. The reason for the considerable decrease in the bandgap was attributed to the upward shift of the valence band of GaN film due to the hybridization of the O_2*p*_-N_2*p*_ orbitals [160].

The similar strategy was used to synthesis 2D InN film via two-step synthesis technique [160]. In this method, the vdW exfoliated 2D In_2_O_3_ film was extracted from the surface of liquid indium. An intermediate bromination step was performed to transfer In_2_O_3_ to InBr_3_. A 20-s post-processing stage of In_2_O_3_ film adjacent to HBr source was enough to complete the phase transformation from In_2_O_3_ into InBr_3_ [61]. The following ammonolysis treatment of InBr_3_ 2D film by NH_3_ source at the temperature of 630 °C resulted in the phase transformation of InBr_3_ into InN [160]. The following characterization studies confirmed the development of crystalline 2D ultra-thin wurtzite InN nanostructures with the crystalline lattice constant of 5.5 Å with the bandgap of 3.5 eV [160]. Generally, the multi-step synthesis technique was found as reliable approach towards the synthesis of advanced 2D nanostructures by using the surface oxide of liquid metals. The developed 2D nanostructures have clearly demonstrated novel electronic and optoelectronic characteristics which are not expected and observed in the similar 2D nanostructures, synthesized via high-vacuum deposition-based methods [164].

#### *SnO/In*_*2*_*O*_*3*_* 2D Heterostructures*

**Fig. 13:**
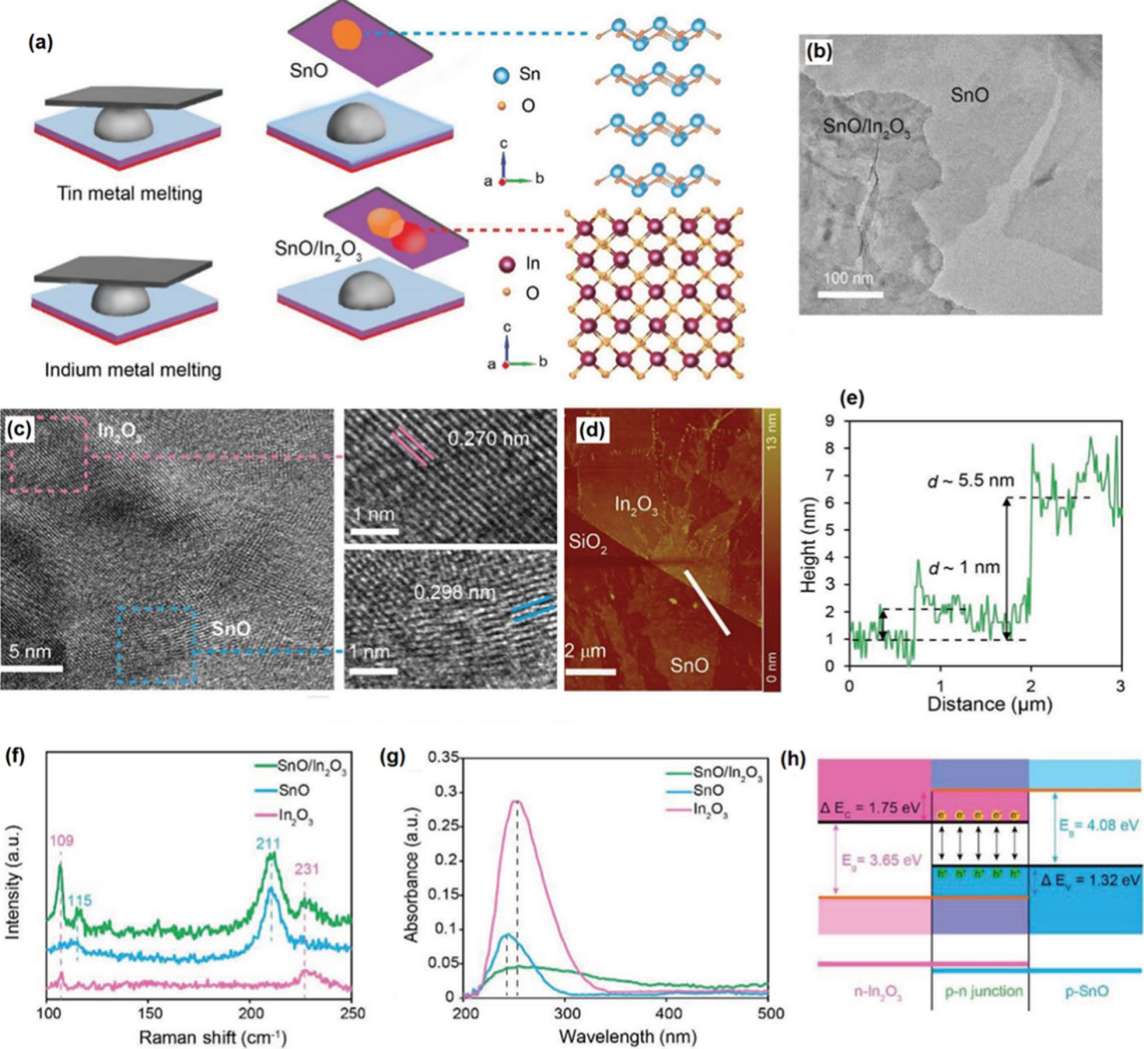
**a** Schematic illustration of fabrication of 2D vdW heterostructured SnO/In_2_O_3_ film. **b** The HRTEM image of heterostructured SnO/In_2_O_3_ films. **c** The HRTEM image of atomic structure of heterostructured films show the high crystalline structure of SnO and In_2_O_3_ film with their lattice spacing distance. **d** The AFM of heterostructured SnO/In_2_O_3_ film with **e** the profile thickness of the films. **f** The Raman spectra of SnO, In_2_O_3_ and SnO/ In_2_O_3_ 2D films. **g** The optical absorption of SnO, In_2_O_3_ and SnO/ In_2_O_3_ 2D films. **h** The band alignment of SnO/ In_2_O_3_ 2D heterostructures. Reprinted with permission from [167]

The *p-n* heterojunctions are now the building blocks of the semiconductor devices where the hetero-interfaces between two semiconductor materials with the different types of charge mobility properties are used to fabricate various types of electronic and opto-electronic instruments. Specifically, the heterostructured devices based on 2D films can be developed by several different approaches and techniques including mechanical exfoliation from the host material, CVD, ALD, molecular beam epitaxy, and pulsed laser deposition [165, 166]. Interesting new strategy was recently employed to fabricate vdW heterostructured 2D layered materials via stacking of various 2D layered crystals extracted from their liquid-based metals and alloys. A *p-n* heterojunction was developed by the vdW exfoliation of 2D SnO and In_2_O_3_ films [167]. In this method, 2D SnO/In_2_O_3_ vdW heterostructures developed [167] by the vdW transfer of surface oxide film of liquid tin (*p*-SnO) on top of the natural surface oxide of indium (*n*-In_2_O_3_). The developed 2D heterostructured film finally composed of *p*-SnO/*n*-In_2_O_3_ 2D films attached on top of each other via vdW forces (Fig. 13a, b) [167]. The following microstructural studies in atomic scale levels confirmed the formation of a vdW heterointerfaces between SnO/In_2_O_3_ films with two crystalline structures with the different characteristics fabricated over Si/SiO_2_ substrate (Fig. 13c). The lattice spacing of 0.27 nm corresponded to the (321) plane of crystalline In_2_O_3_, and the other 2D film with 0.298 Å is attributed to the (101) plane of SnO. The heterostructured film had the thickness of 4.5 nm (Fig. 13d, e). The Raman and optical characterization studies confirmed the development of heterointerfaces with optical absorption peak of SnO/In_2_O_3_ film located at 251 nm, which was a number between 246 nm (SnO absorption peak) and 256 nm (In_2_O_3_ absorption peak in Fig. 13f, g) [167]. One of the main properties of heterostructured film was strong interlayer coupling of charge carriers which affected the optical absorption of 2D film. The bandgap of heterostructured film was narrowed to 2.30 eV while the bandgap of individual SnO and In_2_O_3_ were 4.8 eV and 3.65 eV, respectively, [167]. The schematic of band alignment at 2D SnO_2_/In_2_O_3_ vdW hetero-interfaces was demonstrated at Fig. 13h. The effective bandgap at *p–n* junction was expected to be 2.30 eV, which was close to the calculated value of band gap of 2D heterostructured film from Tauc plot. The narrow bandgap assisted the charge separation at hetero-interfaces, thus the photoexcited electron–holes can be separated easily and they can feasibly migrate from the valence band of SnO to the conduction band of In_2_O_3_ film. The specific energy band alignment at the hetero-interfaces of vdW SnO_2_/In_2_O_3_ demonstrated quite impressive capabilities and excellent photodetectivity of 5 × 10^9^ J with the photocurrent ratio of 24 and the outstanding photoresponsivity of 1047 A/W [167]. Here, the liquid metal printing technique was found as the valuable potential approach for fabrication of heterostructured 2D materials, which do not naturally exist as the layered structures in nature.

Therefore, the natural ultra-thin metal oxide films on the surface of liquid metals and alloys were found as functional materials with tremendous applications. Ultra-thin nature, pin-hole free and considerable lateral dimensions of these films accompanied by the facile vdW delamination of them on desired substrate provided a great platform for successful synthesize of novel generation of 2D materials and related electronic instruments [168]. Table 4 has provided a representative information on the recently developed 2D materials and devices based on 2D surface oxide film of the liquid metal and alloys.

**Table 4:** Summary of properties of 2D surface films of liquid metals. Reprinted with permission from [168]

2D film	Based alloy	Synthesis method	Specifications
Ga_2_O_3_	Gallium Galinstan (EGaInSn)	vdW exfoliation (Touch printing) Gas injection in aqueous media	Crystalline (Exfoliation) Amorphous (gas Injection)
Al_2_O_3_	Galinstan-Al	vdW exfoliation (Touch printing) Gas injection in aqueous media	Crystalline (Exfoliation) Amorphous (gas Injection)
Gd_2_O_3_	Galinstan-Gd	vdW exfoliation (Touch printing) Gas injection in aqueous media)	Crystalline (Exfoliation) Amorphous (gas Injection)
HfO_2_	Galinstan-Hf	vdW exfoliation (Touch printing) Gas injection in aqueous media	Crystalline (Exfoliation) Amorphous (gas Injection)
SnO	Tin (Sn)	vdW exfoliation (Touch printing)	Crystalline, p-type, ambipolar characteristics
Bi_2_O_3_	Bismuth	vdW exfoliation (Touch printing), oxidation in controlled atmosphere	Crystalline
SnO/In_2_O_3_	Tin (Sn)-Indium (In)	Sequential vdW exfoliation (Touch printing)	Crystalline, p-n heterojunction
SnO_x_	Tin–Bismuth	Oxygen gas injection into Sn-Bi alloy covered by non-aqueous solvents	SnO_x_ amorphous nanoflakes and SnO_x_ crystalline nanorods
MnO_2_	Gallium-Indium (EGaIn)	Galvanic replacement of galinstan particles by MnO_4_^−^	Metallic core/2D MnO_2_ shell Crystalline
MnO_2_	Gallium-Indium (EGaIn)	Galvanic replacement of galinstan particles by MnO_4_^−^	Metallic core/2D MnO_2_ shell Crystalline
MoS_x_	Galinstan	Cathodic reaction in aqueous solution containing the (NH_4_)_2_MoS_4_	Crystalline
Cu_x_O	Galinstan	Chemical interaction of Galinstan in NH_4_OH + CuSO_4_ solution	Morphology dependence to PH content (from crystalline nanosheets to dendrites)
TiO_2_	Gallium-Ti	Metal alloying, Gas injection in aqueous media	Crystalline, Wrinkled nanosheets
GaPO_4_	Gallium	vdW exfoliation (Touch printing) Reaction with Phosphorous at 350 °C (N_2_ + H_3_PO_4_)	Crystalline
GaN	Gallium	vdW exfoliation (Touch printing) Ammonolysis reaction	Crystalline, high carrier mobility of 21.5 cm^2^V^−1^ s^−1^
InN	Indium	vdW exfoliation (Touch printing) Bromination + Ammonolysis reactions	Crystalline
SnS	Stanium	vdW exfoliation (Touch printing) Reaction with H_2_ at 600 °C	Crystalline
Ga_2_S_3_	Gallium	vdW exfoliation (Touch printing) Reaction with H_2_S at 600 °C	Crystalline
Bi_2_S_3_	Bismuth	vdW exfoliation (Touch printing) Reaction with H_2_S at 450 °C	Crystalline
In_2_S_3_	Indium	vdW exfoliation (Touch printing) Reaction with H_2_S at 450 °C	Crystalline with sandwiched structure
WS_2_/Ga_2_O_3_	Galinstan	vdW exfoliation (Touch printing) and transfer of 2D Ga_2_O_3_ film on the surface of monolayer WS_2_	Passivation of monolayer WS_2_ via glassy Ga_2_O_3_ 2D film

### Novel Applications of 2D Semiconductors

#### Memristors Based on 2D Semiconductors

The information technology today faces the fast growing of amount of generated data. The data processing in new era requires development of advanced data storage technologies far beyond of concept of current silicon-based technology. Today Si-based memories are facing their technological limits, furthermore, the complementary CMOS instruments are also suffering from the physical dimensional retractions and technological problems [169]. Consequently, it is highly required to develop new nanostructured materials with improved properties and also to modify the existing devices to circumvent the technological restrictions related to the storage capacity of memory units. The von Neumann technology tries to emulate the working mechanism of human brain where the complicated networks of interconnected biological synapse enable us to memorize, learn, remind, recognize, process and understand the multi-task operations with minimum energy consumption. An average power consumption for a singular synaptic event is in the range of 1–10 fj, which is at least 5 orders of magnitude lower than the energy consumption of the existing conventional computers [170]. The current von Neumann technology architecture is not efficient since their memory and processing units are physically separated. A low-energy consumption non-volatile memristors with nanoscale dimensions, high performance, and improved processing speed can respond the current memory technology which is in its bottle-neck stage [170]. Regarding the energy consumption issues, 2D materials can be employed as ultra-thin nanostructural memristors. The resistive switching (RS) behavior of 2D materials has also been explored recently. It was found that the employment of 2D materials in memory units could potentially tackle the current limitations in memory devices [171]. Recently, the nonvolatile characteristics of 2D materials have been extensively investigated and characterized. The 2D semiconductor layered materials and ultra-thin oxide films possess a wide range of properties in various fields which also can be accompanied by their memristive characteristics.

#### Memristors Based on TMDCs

**Fig. 14:**
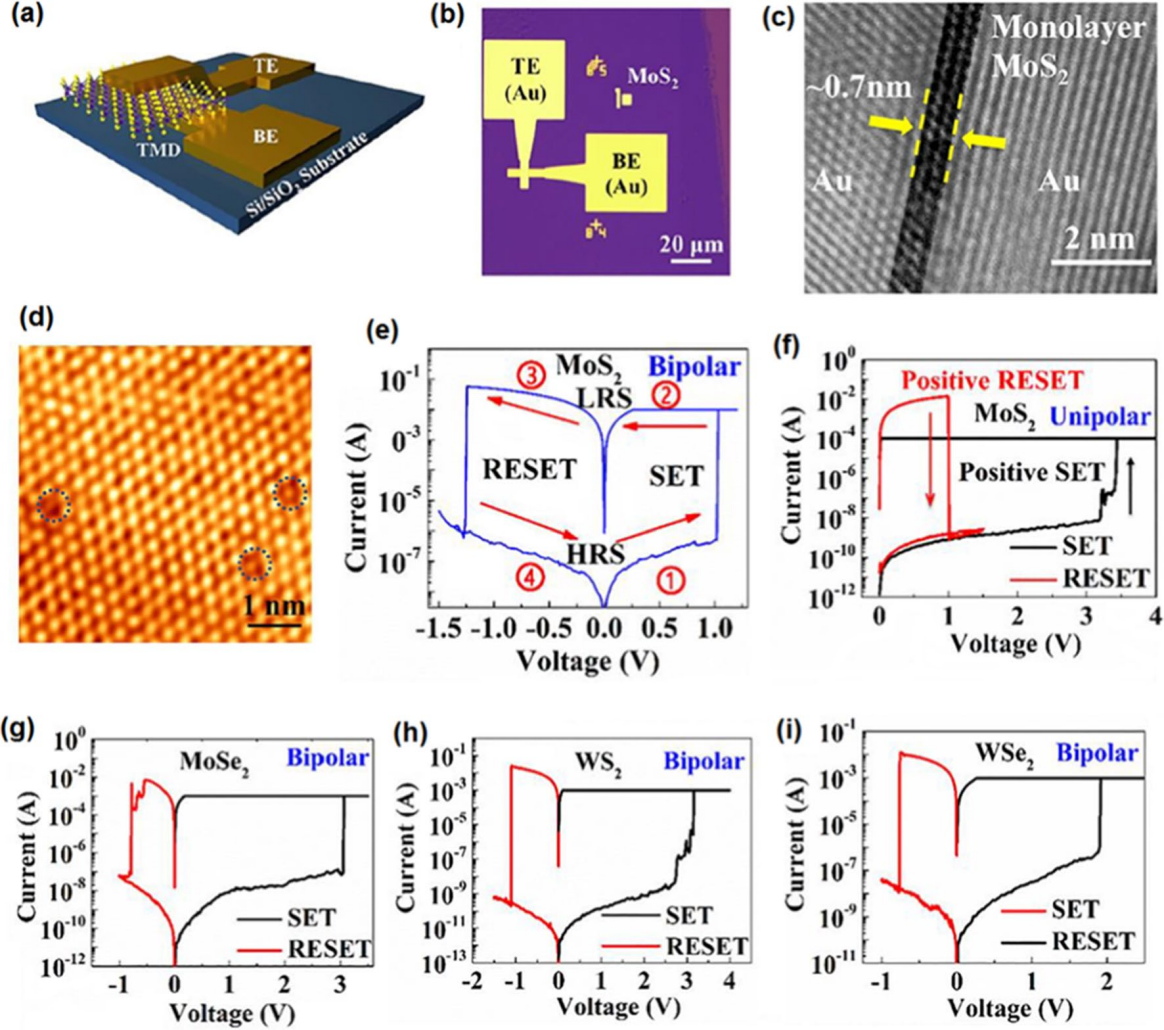
**a** Schematic interpretation of metal/insulator/metal atomristor device based on TMDCs monolayers, **b** the corresponding optical image of device and the **c** cross-sectional TEM image of Au-Monolayer MoS_2_-Au device. **d** The atomically resolved STM image of monolayer MoS_2_ film deposited on Au electrode. The sulfur vacancies indicated by dashed lines. **e** The typical *I-V* curve of atomristor device based on monolayer MoS_2_ monolayer device. The typical bipolar RS behavior is observed. **f** The occasional unipolar RE behavior of device in monolayer MoS_2_ device. **g**, **h** and **i** graphs respectively demonstrate the typical bipolar RS behavior of atomristor devices based on monolayer MoSe_2_, WS_2_ and WSe_2_ films. Reprinted with permission from [172]

The recent development in nanodevice fabrication technology enabled the employment of ultra-thin TMDC films in functional instruments. The CVD and metal organic CVD were successfully employed to deposit monolayer MoS_2_, MoSe_2_, WS_2_, and WSe_2_ 2D films in a sandwiched in a vertical configuration between Au electrodes over Si/SiO_2_ substrate (Fig. 14) [172]. It was confirmed that monolayer TMDC films had no grain boundaries and they are empty of oxide compounds. Thus, an oxide free hetero-interface was shaped between Au electrodes and 2D TMDC films. This sharp and clean hetero-interface increased the possibility of precise measurement of electrical properties of 2D films. The graphical scheme of the memristor devices, the top view of device and the cross-sectional TEM image of Au/MoS_2_/Au memristor are presented in Fig. 14a–c, respectively. The characterization of 2D MoS_2_ by atomic resolution scanning tunnelling microscope (STM) (Fig. 14d) demonstrated relatively high quality and additionally showed local sulfur vacancy defects in the MoS_2_ film. The unit based on monolayer MoS_2_ film verified high *on*/*off* ratio above 10^4^ and the corresponding retention time as high as 10^6^ s. The bipolar resistive switching was observed in the behavior of all memristors based on MOS_2_, MoSe_2_, WS_2_, and WSe_2_ 2D films. Both unipolar and bipolar resistive switching behavior were detected during the study of resistive switching response of MoS_2_ films. The abrupt increase of the device current in all samples indicated the occurrence of dielectric break down phenomenon. The formation of conductive filaments (CFs) in the 2D active layer of memristors was the main mechanism of RS (Fig. 14e–i). Thus, the electroforming can be avoided by scaling down the thickness of active memristor layer into the few nanometer range. The main drawback of this strategy is the excessive leakage current originated from the trap-assisted device tunnelling [165]. Occasionally, the unipolar RS was observed in the certain single-layer MoS_2_ metal/insulator/metal units as was shown in Fig. 14 f. The typical bipolar RS behavior was also presented in the study of resistive behavior of atomristors based on monolayer MoSe_2_, WS_2_ and WSe_2_ films, respectively [172].

**Fig. 15:**
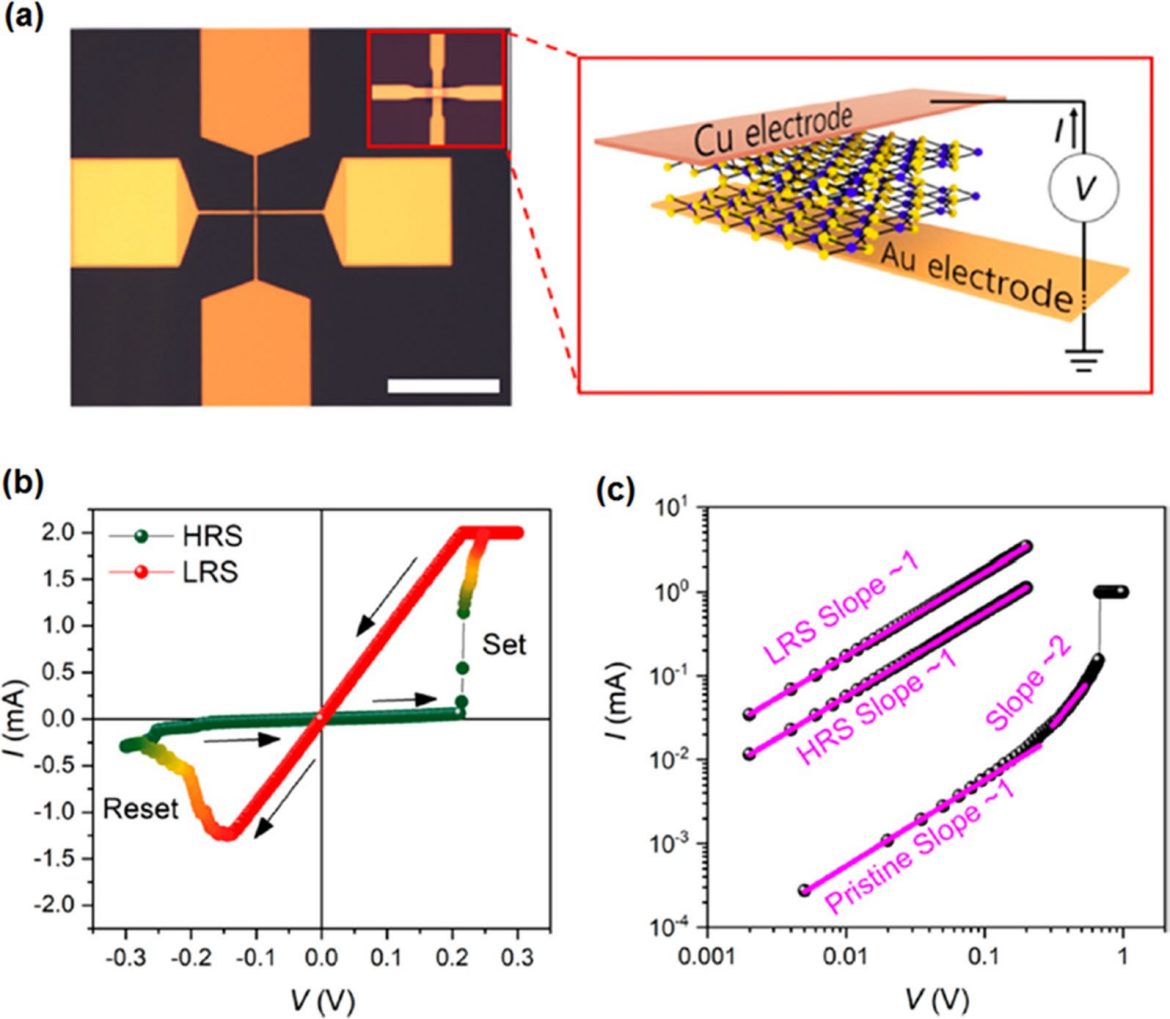
The optical image of Cu/MoS_2_ double layer/Au memristor device with scale bar of 100 μm accompanied by the graphical scheme of device with top Cu electrode and bottom Au electrode. The memristor’s crossbar area is 2 × 2 μm^2^. **b** The *I-V* curve of memristor device with bipolar RS with the 0.25 V Set voltage and − 0.15 V Reset voltage. **e** The logarithmic scale *I*–*V* curve of device for the pristine state before and after forming process, and for LRS and HRS after the forming process. Reprinted with permission from [173]

In another configuration, a memristor based on monolayer MoS_2_ was developed where a Cu electrode was employed as the active electrode to fabricate electrochemical metallization memory device [173]. In this instrument, the Au film play the role of inert conductive electrode. The scheme of this device is demonstrated in Fig. 15a. Due to the diffusion of Cu ions in MoS_2_ layer a conductive atomic-scale Cu filament is formed in the MoS_2_ double monolayer. Therefore, this memristor falls under the category of electrochemical metallization memories (ECM), whereas the memristive performance of other 2D MoS_2_ device with the inert electrode (similar Au) is based on migration of the sulfur vacancies. The sulfur vacancies in MoS_2_ thin films make conductive filaments with atomic scale dimensions, and hence these types of MoS_2_ memristors are known as the valence change memories (VCM) [173].

In fabricated ECM (Fig. 15), the two-dimensionality and electrochemical metallization by Cu cationic filaments resulted in the considerable decrease in switching voltage down to 0.2 V (Fig. 15b). This characteristic has arisen from the lower diffusion activation energy of Cu cations in MoS_2_ layer compared with that of sulfur vacancies in 2D MoS_2_ film [173]. The bipolar resistive switching was the main characteristics of the *I-V* curve of memristor with a set voltage at 0.25 V and a reset voltage at − 0.15 V. It was observed that the majority of double-layer MoS_2_ memristors are free of the forming process. By using a double-layer MoS_2_ memristor the transport mechanism in Cu/double layer MoS_2_/Ag device was investigated. Figure 15b depicts the *I–V* characteristics of memristor before the forming process (pristine state) as well as for HRS and LRS (after forming). The instrument performed in pristine state with the resistance of 16.6 kΩ when the applied voltage on the unit was less than 0.3 V. Quantum tunnelling was recognized as the conduction mechanism in memristor in the pristine state. In this condition, the resistance is much larger than the resistance of device after the forming process of memristor. The further increase of applied voltage beyond the 0.3 V in pristine state is accompanied by the transition in *I–V* curve into the slope 2 region (*I ∝ V*^*2*^), which corresponds to the space-limited conduction states. The dramatic increase of current after increasing of applied voltage (further than 0.6 V) is attributed to the formation of Cu conductive filaments in MoS_2_ film (Fig. 15c). Higher imposed voltage on Cu electrode resulted in the oxidation of Cu top electrode. This resulted in the migration of Cu cations toward the bottom Au electrode to reduce and deposit there. The conductive Cu filaments were built up along the defective grain boundaries of 2D MoS_2_ film and the device switched to LRS.

#### Complementary Resistive Switching in Transition Metal Oxide Films

**Fig. 16:**
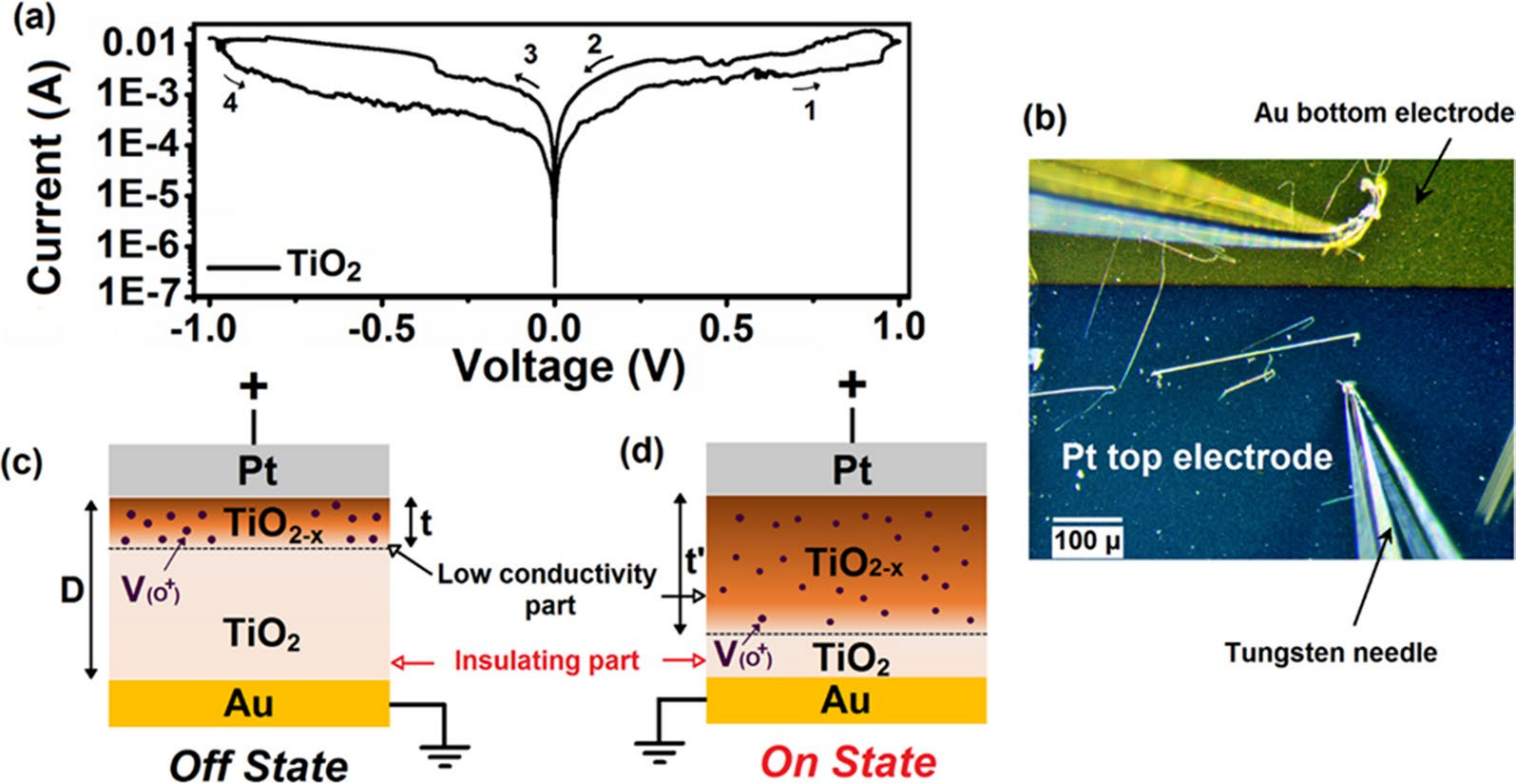
**a** Typical logarithmic scale depiction of *I–V* sweeping graph of Pt/In-doped TiO_2_/Au memristor. **b** Device is set off, the metallic cations are distributed in top layer of In-doped TiO_2_ film. **c** The formation of filamentary conductive channels, the device is set On (Set, stage 1). **d** The depletion of metallic cations adjacent to the bottom electrode. The device is set off again (Reset, stage 2). **e** By imposing the reverse gate voltage, the metallic cations again move toward the cathode electrode (top Pt electrode) and again form a metallic cation bridge and the device is set On (Set-stage 3). **f** By depletion of metallic cations, at Pt electrode the device is set off again (Reset, stage 4). Reprinted with permission form [174]

The ion doping of semiconductor films can tangibly alter the charge transfer mechanism in these ultra-thin films. The memristive characteristics of 2 semiconductors are also tangibly affected by the presence of other foreign ionic species inside active materials of memristors. TiO_2_ is one of the well-known recognized semiconductors with resistive switching characteristics. A 7.0-nm-thick TiO_2_ ALD film was used to investigate the impact of ion intercalation in resistive switching of this ultra-thin film [174]. To understand the effect of Indium (In) intercalation in active layer of memristor (TiO_2_), an ion incorporation set up was made. The ion intercalation in this set-up was facilitated by imposed driving voltage on the ion-containing solution covering the surface of 2D TiO_2_ film. The potentiodynamic measurements (*I–V* sweep) of Pt/TiO_2_/Au and Pt/In-doped TiO_2_/Au memristor under the dark condition showed different RS mechanisms for In-ion doped and non-doped TiO_2_ films. Pt/TiO_2_/Au device shows bipolar switching behavior (Fig. 16a). The *V*_Set_ = 0.94 and *V*_Reset_ = − 0.98 during *I–V* sweeping measurement were observed [174]. A bipolar RS behavior of film was explained by the ionic drift of oxygen vacancies owning to employed voltage on the electrochemically inert Au and Pt electrodes. This unit is categorized as VCM cells. The schematic representation of oxygen vacancies distribution for both *off* and *on* states are shown in Fig. 16b and c. The dielectric member of memristor is characterized with two parts: low conductive TiO_2-x_ with the thickness of *t* and insulating part with the thickness of *D-t*. The applied voltage on the electrode caused the ionic drift of oxygen vacancies, which is accompanied by the shift of border between low conductivity component and insulating part of active layer of memristor. In this condition, the *I–V* curve of device is not further Ohmic and will be non-linear. In this proposed model, the thickness of insulating layer should be small enough (few nanometer) to let the electric field develops and promotes the ionic drift of defects [174].

**Fig. 17:**
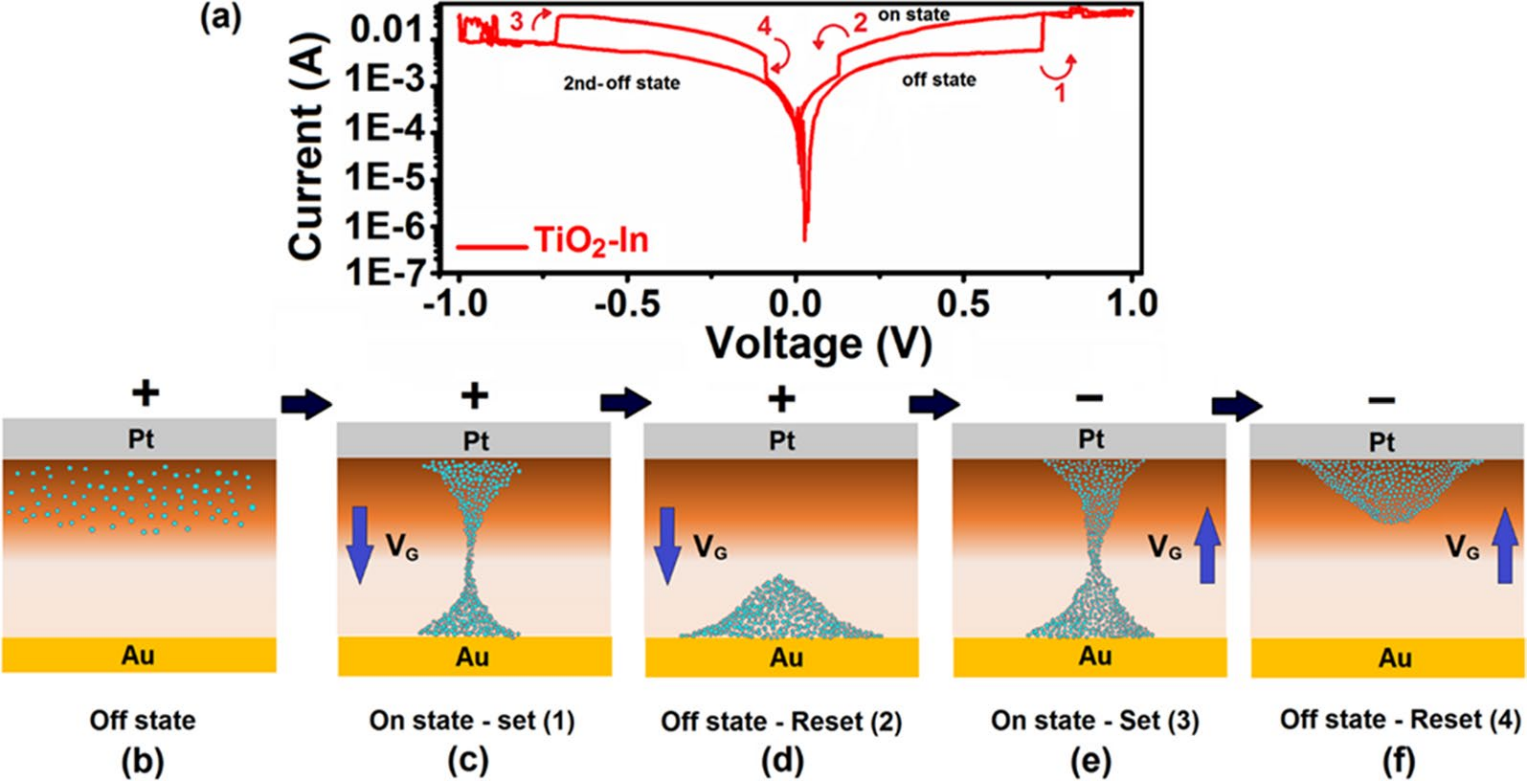
The Pt/TiO_2_/Ti/Pt stacked crossbar cells: **a** the SEM top view of (60 nm)^2^ cross-point, **b** The TEM cross-sectional image of 100 nm^2^ Pt/TiO_2_/Ti/Pt device with **c** its corresponding schematic graph of the plug and disc region in the TiO_2_ layer. Reprinted with permission from [174]

The In-doped TiO_2_ film demonstrated a complementary resistive switching (CRS) behavior during the voltage sweeping between − 1.0 V and + 1.0 V (Fig. 17a) device is set at a higher voltage and again reset at another lower voltage of the same polarity [174]. The cell was set for the first time at *V*_Set1_ = 0.732 V and then reset again in the same polarity at *V*_Reset1_ = 0.1277 V. In the negative voltage, the second LRS to HRS switching occurred at *V*_Set2_ = − 0.7055 V and again reset to HRS at *V*_Reset2_ = − 0.0901 V. Thermally assisted charge transfer mechanism is the most possible theory which explain the resistive switching in the ion-doped TiO_2_ 2D film. The unipolar switching confirms the filamentary nature of resistive switching. The filamentary resistive switching is mostly based on the ECM of the mobile ionic species caused by the migration of metallic cations. It facilitates the development of filamentary metal bridges (*on* state), which can be ruptured later (*off* state). The filamentary mechanism is the characteristic of this memristor, which is fabricated with electrochemical active electrodes, while the In-doped TiO_2_ memristor was sandwiched between inert Au and Pt electrodes. It is expected that the switching behavior of In-doped TiO_2_ device was originated from the migration of both anion and cation components. The versatile switching behavior of In-doped TiO_2_ device can prove that both anion and cation components are involved in CRS mechanisms. The proposed model suggested that the redistribution of oxygen vacancies and Indium ions inside TiO_2_ layer can affect, alter and facilitate the RS. In the heterostructured oxide stacks, the conjunction of an oxygen deficient oxide layer (LRS mode) with another oxygen rich layer (HRS mode) triggers the CRS of heterostructured memristor which is caused by the movement of oxygen vacancies. This gradual change of oxygen concentration can trigger occurrence of CRS of memristor due to the difference in resistance of TiO_2_ stack. From another point of view the formation of filamentary conductive channels assist the RS and increase the conductance of devise. At the initial state or *off* state (Fig. 17b), the top In-doped layer is in LRS mode while the bottom layer is in HRS. By applying the threshold voltage, the ionic species migrate into lower insulating layer resulting in the formation of conducting filaments and paths (Fig. 17c) and then device reaches to its *on* state (Set 1). At higher positive voltage, the ionic species in upper layer (mostly In-ions) are depleted (Fig. 17d) and again device will reach to its *off* state (Reset 2) and demonstrates HRS behavior. The similar phenomenon is expected to occur at the negative biases in which the first *on* state (Fig. 17e) switches the memristor to new LRS, following another *off* state after depletion of the ionic species (Fig. 17f). The versatility of CRS behavior of all samples was repeatedly tested several times and the same behavior was observed.

### Artificial Optical Synapses Based on 2D Semiconductors

**Fig. 18:**
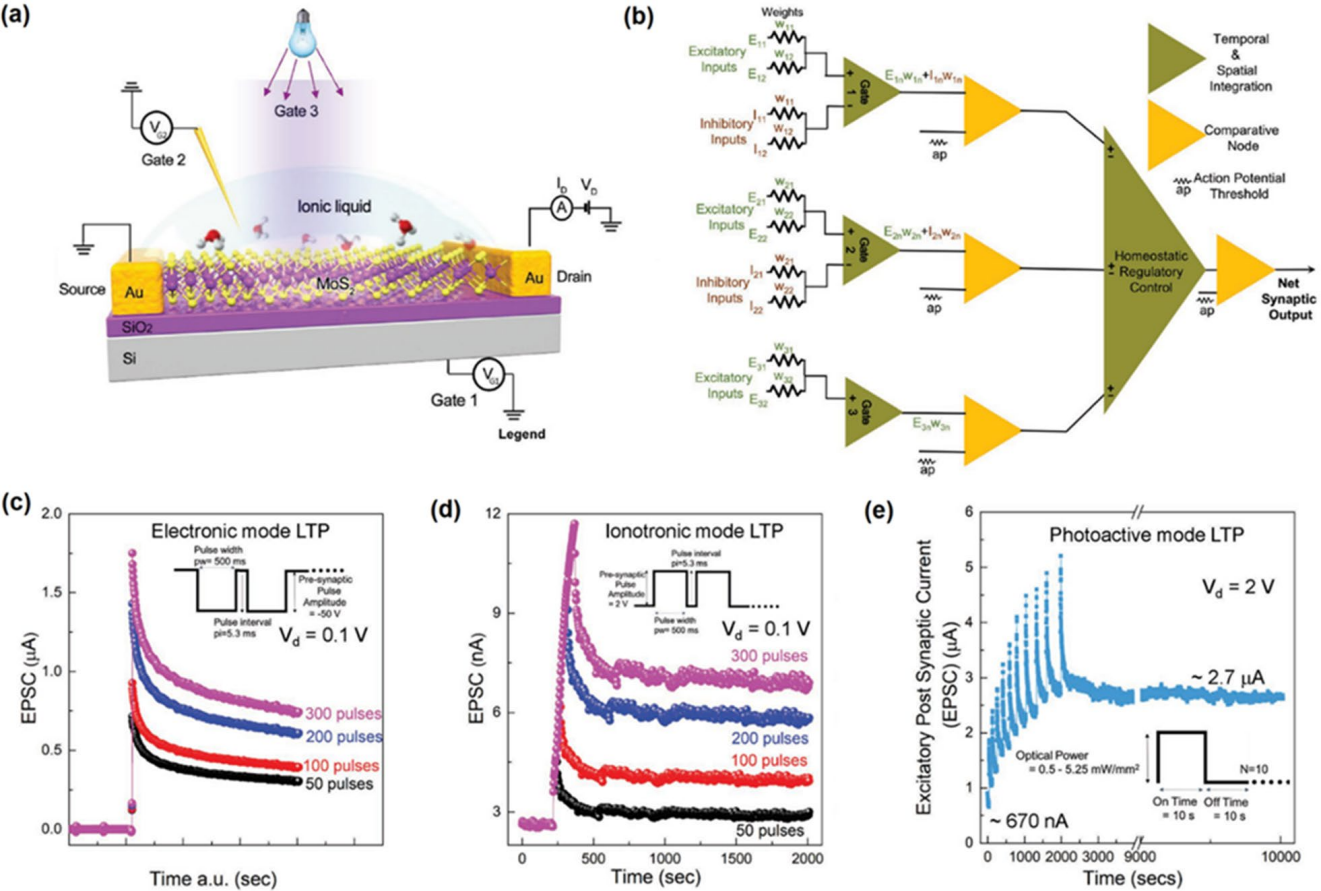
Graphical scheme of triple modulated architecture of artificial 2D MoS_2_ synaptic device. **b** The analogue circuit depicting the interplay between the various parameters for stimulation of synaptic plasticity. **c** The interplay between the Hebbian and synaptic plasticity in MoS_2_-based 2D synaptic device, which depicts the enhancement in the strength of long-term potentiation functionalities of device as he function of training by electronic-mode, **d** Ionotronic mode and **e** photovoltaic mode. In photovoltaic mode, the light intensity is altered (from 0.5 to 5.25 mW mm^−2^) to enable training by optical pulses. Reprinted and reproduced with permission from [178]

The human brain operation is highly energy efficient. The data storage and processing are functioned in brain with the lowest possible energy consumption. This ultra-low energy consumption is inspired by the functionalities of biological nervous system. Based on the brain performance, a novel type of computing and processing system was proposed by Alan Turing in 1948, when he introduced the idea of using a computing device with the interconnected network of artificial neurons [175]. It is envisaged that the interconnect network of artificial synapses can effectively solve and overcome the restrictions of the traditional van Neumann computing. The biological synapses, as one of the key members of the neural system, are the information channels ensuring short-term computation, long-term learning and memorization by tuning the synaptic weights [176]. As a biological structure, a chemical synapse is a gapped connection between two neurons through which the communication between two individual axons is created by biochemical reactions. The synapse is a biological junction where the electrochemical waves (action potentials) are transmitted through the axons of neuron. The energy consumption of a biological synaptic event is tangibly lower than that of any other similar device. Analogously, an artificial synapse is a unit which mimics the behavior and performance of the biological synaptic junction to transfer the synaptic signals. From the technical point of view, memristors are the best candidates capable of successful emulation of the characteristics of a biological synapse [177]. The resistive switching mechanism is the basic principle of the memristors. A sandwiched insulator or transition metal oxides between two conductive electrodes is the typical structure of an artificial synaptic device. The synaptic properties are observed in variety of materials with different resistive and synaptic mechanisms. In fact, the diversity of materials with synaptic characteristics experiences a rapid progress, and thus the fundamental understanding of related synaptic mechanisms is currently progressing. Metal oxide resistive random access memories (RRAMs) (such as TiO_2_, AlO_*x*_, HfO_*x*_, WO_*x*_, TaO_*x*_), conductive bridge synapses (Ag- and Cu-based electrodes), phase-change material-based synapses (PC synapse), ferroelectric material-based synapses, magnetoresistive (MR)-based synapses, carbon nanotube based synapses, and organic nanostructured-based synapses are some of the active memristor materials that demonstrated attractive synaptic characteristics [177]. The optical stimulated synaptic devices are recently introduced into telecommunication technology. In optical synapses, optical pulses of light stimulate the generation of charge carriers between two conductive electrodes. The photogenerated charge carriers in atomically thin 2D MoS_2_ film allow the modulation of the synaptic weights with photonic pulses. This optical capability of artificial synapse is highly important that capitalizes the possibility of high-bandwidth optical communication protocols. The schematic of 2D MoS_2_-based synaptic unit is depicted in Fig. 18a [178]. As the main privilege, the incorporation of the optical and electrical pulses addressed the different charge trapping mechanisms to precisely modulate the synaptic weights. Furthermore, the instrument was capable of neuromodulation to achieve the plasticity and metaplasticity properties via dynamic control of spike-time-dependent plasticity (Hebbian theory). The schematic of analogue circuit is demonstrated in Fig. 18b depicting the interplay between Hebbian and homeostatic plasticity. The interplay between Hebbian and homeostatic synaptic plasticity in MoS_2_ synapse is provided in Fig. 18c–e, respectively. The long-term potentiation strength was achieved by employment of sequential voltaic training pulses in synaptic instrument. It was observed that the synaptic weights increase linearly by increasing the number of training pulses. It was also demonstrated that the application of 100 training pulses resulted in the weight change of synapse up to 117% in electronic mode, while the same number of training pulses in ionotronic-mode resulted in the increase of weight up to 223%. (Fig. 18c, d) [178]. In the optically stimulated synaptic unit, a strong long-term potentiation was observed in the 2D MoS_2_ artificial synapse via using the optical gating function (Photoactive mode). The light pulses (*λ* = 445 nm) played the role of presynaptic signals. Up on the illumination on the surface of 2D MoS_2_ synapse, the conductance increased rapidly and then continued until the gradual saturation when the conductance was one order of magnitude above the value of dark-current. A rapid drop of conductance occurred on the termination of optical illumination source, but the conductance still remained in high values (Fig. 18e). The band-to-band transition is expected to be the main reason for rapid transition, while the long-lasting high conductance values in optoelectronic mode were attributed to the defect or trap-centred slow recombination of generated electron/holes. The observations are in accordance with the proposed model of random local potential fluctuation (RLFP). This state is called persistent conductivity [178]. By the increase of optical pulses and higher number of photons, the decay time became slower. This is consistent with the RLPF model where higher carrier numbers occupy sites of the local potential minima, and therefore, the rate of election/hole recombination would be slower. The vivid manifestation of this phenomenon is slower degradation of photogenerated current in synaptic device.

**Fig. 19:**
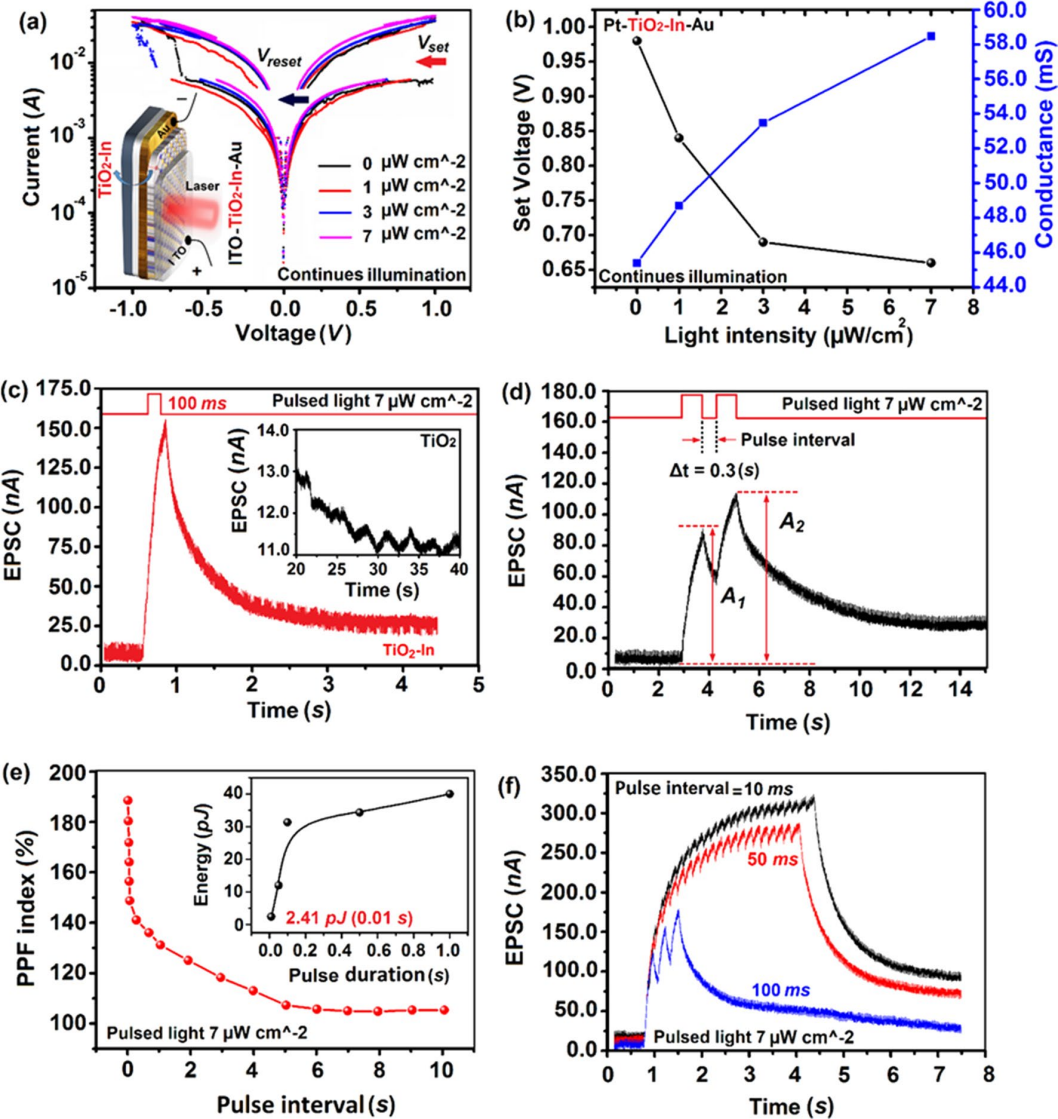
The optical synaptic characteristics of In-doped ultra-thin TiO_2_ films. **a** The logarithmic scale *I–V* curves of ITO/In-doped/Au synaptic device. Variation of the set voltage and conductance vs changes of light intensity for pulsed lasers. **c** The EPSC of synaptic device induced by 7 μW cm^−2^ laser pulse. **d** The PPF of device. **e** The variation of PPF index vs. the pulse intervals. Inset shows the variation of energy consumption vs. pulse duration. **f** The variation of EPSC of synaptic device stimulated with the pulsed light with different pulse intervals. Reprinted with permission from [174]

Two-terminal optical synapse based on In-doped TiO_2_ ultra-thin film was also fabricated for emulation of the synaptic functionalities [174]. The In ions were doped into ~7.0-nm-thick TiO_2_ film deposited by ALD technique. It was observed that the ion doping affected the RS mechanism and also extended the optical absorption edge of ultra-thin 2D TiO_2_ film to the visible light range. It was measured that the set voltage of the TiO_2_-based synaptic device was shifted to the lower voltages after optical stimulation of In-doped TiO_2_ memristor. These observations (Fig. 19a) confirmed the impact of photogenerated electron/hole on occupation of trapping sites in amorphous TiO_2_ film and consequently resulted in the decrease of the set voltage (Fig. 19b) and the increase of memristors’ conductance (Fig. 19b). Furthermore, the increase in light intensity slightly affected the set voltage of memristors, which is an indication of saturation state in the number of photogenerated charge carriers. Therefore, it does not let the device to achieve the lower *V*_Set_ values. The emulation of optical synaptic characteristics was verified by the observation of unrested postsynaptic current caused by singular optical pulse (Fig. 19c). PPF synapse values were demonstrated by two consecutive EPSC spikes with pulse intervals of 0.3 s (Fig. 19d). The rapid decay of PPF index of device [the ratio of amplitude of the 2nd EPSC (A_2_) to the 1st EPSC (A_1_)] was discovered after the increase of pulse intervals. It shows the sensitivity of short-term plasticity of optical synapses to the sequence of optical pulses (Fig. 19e). The calculated energy consumption of In-doped TiO_2_ optical synaptic device for a singular pulse was estimated to be around 2.41 pJ (pulsed optical signal with 10 ms duration) (Fig. 19e). It was observed that the energy consumption of In-doped ultra-thin TiO_2_ optical synapse was highly efficient [174]. Moreover, when the successive laser pulses with different frequencies were employed, transition from STP to LTP emulated by the same optical synaptic unit (Fig. 19f). These results clearly demonstrated that the optical stimuli with the lower pulse intervals are beneficial for facilitation the LTP capabilities. Observations also confirmed that shorter pulse intervals resulted in higher gain values consistent with the effect of residual generated carries on the following pulses [174]. The EPSC and conductance saturation were also detected and observed during the study of synaptic behavior of artificial synapses. This phenomenon was attributed to the saturation of photogenerated electron/holes. It was also shown that the instrument was capable of emulation of bidirectional analogue switching.

### Artificial Optical Nociceptors Based on 2D Semiconductors

**Fig. 20:**
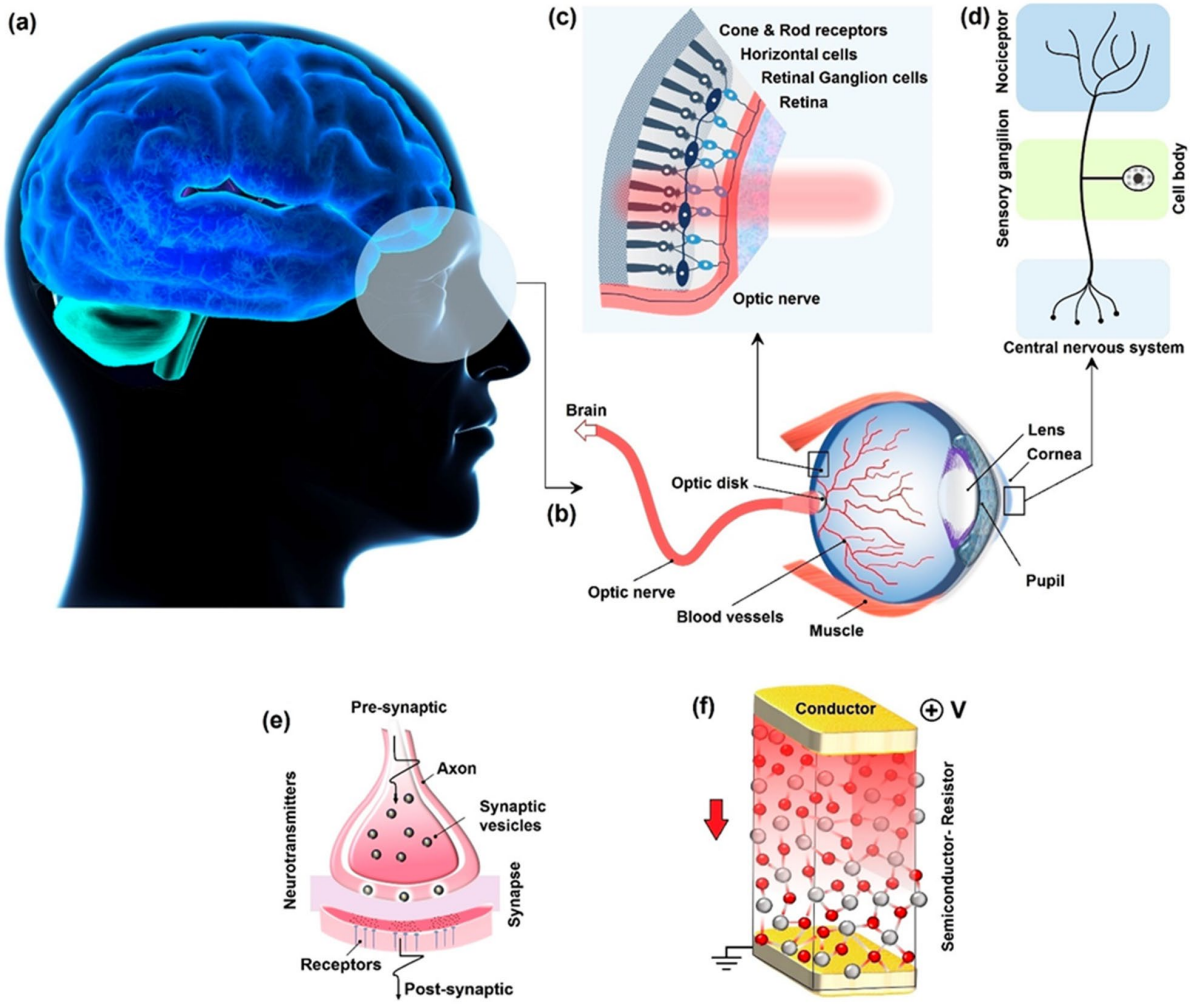
Scheme of human eye receptor and nociceptor system. **a** The human brain as decision making unit receives the informative signals from **b** human eye and its sensory components including the **c** light receptors and nociceptors in Retina section. **d** Shows a typical nociceptor with its components. **e** Is the typical schematic representation of a natural synapse and **f** its artificial counterparts in conductor/ semiconductor /conductor sandwiched configuration. Reprinted with permission from [52]

Mimicking the human brain functionalities by using neuromorphic-based technologies is quite essential achievement towards the development of the artificially engineered bio-inspired electronic instruments [52]. The emulation of human sensory system and the sensorimotor functionalities are significant hurdles in biomimetic studies. Visual processing is fulfilled by outstanding features of the human’s eye (Fig. 20a) [52]. Eye, as the natural visual detector and processor consists of a large number of receptors and nociceptors (Fig. 20b–d). In fact, nociceptor is a key sensory receptor that recognizes noxious stimuli, which in turn generates and delivers the warning signals to the central nervous system. The cornea has the highest number of nociceptors in the human eye and the majority of the corneal nociceptors are polymodal. Looking at the nervous system, the biological synapse is the fundamental base of sensorimotor system facilitating various functionalities including the pain signal transfer in the neural system (Fig. 20e). The analogous artificial nociceptive device with similar synaptic functionalities is composed of a semiconductor ultra-thin film sandwiched between two conductive layers (Fig. 20f).

**Fig. 21:**
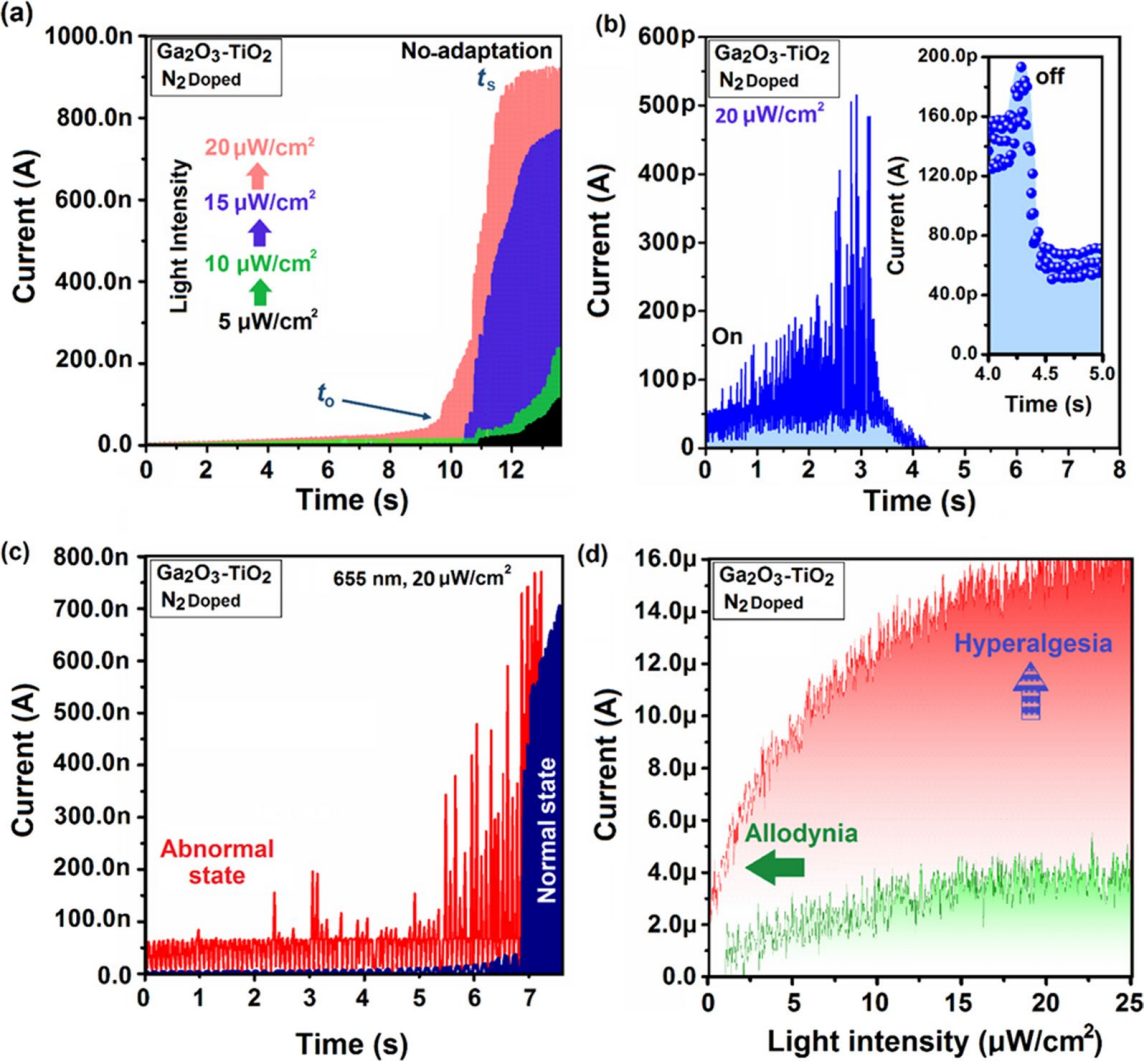
**a** The photoinduced nociceptive behavior of Ga_2_O_3_ (N_2_)/TiO_2_-based heterostructured devices. The effect of light intensity (*λ* = 655 nm) on threshold time (*t*_0_) and saturation time (*t*_s_) of Ga_2_O_3_ (N_2_)/TiO_2_ nociceptor. The light frequency was 20 Hz. **b** The relaxation characteristics of Au- Ga_2_O_3_ (N_2_)/TiO_2_-ITO nociceptor. The light frequency was 20 Hz. **c** The distinction of photoresponse behavior of Au-Ga_2_O_3_ (N_2_)/TiO_2_-ITO nociceptor in normal and abnormal state. The light frequency was 20 Hz. **d** The allodynia and hyperalgesia behavior of Au-Ga_2_O_3_ (N_2_)/TiO_2_-ITO opto-nociceptor. Reprinted with permission from [179]

Optical artificial nociceptors built upon ultra-thin amorphous all-oxide heterostructures with ionic transport, anisotropic electrical characteristics and ultimate transparency. A heterostructured 2D Ga_2_O_3_/TiO_2_ film was fabricated to make an optical nociceptor device [179]. To alter the heterointerfaces between Au electrodes and TiO_2_ optical semiconductor films, the Ga_2_O_3_ 2D film was annealed in N_2_ atmosphere. The successful incorporation of N_2_ atoms into ultra-thin Ga_2_O_3_ film was accompanied by phase transformation of Ga_2_O_3_ into more conductive phase. Raman spectroscopy measurements showed the characteristic peak of Ga-N bonding after annealing process in N_2_ atmosphere [179]. The Ga_2_O_3_ film was still in high level of transparency (up to 90%) [179]. From the PL spectra, it is found that the bandgap of 2D WO_3_ is around 2.88 eV. The PL spectra also showed notable modification of bandgap after RTA of Ga_2_O_3_ 2D film in N_2_ atmosphere. The bandgap of 5-nm-thick Ga_2_O_3_ film was shifted from 4.1 eV to 3.46 eV for N_2_ doped Ga_2_O_3_ film. It was observed that the oxygen vacancies were replaced by atomic nitrogen in the Ga_2_O_3_ nanostructure. The heterointerface engineering of 2D Ga_2_O_3_ (N_2_)/TiO_2_ altered the resistive switching mechanism of 2D heterostructured memristor film [179]. A self-rectifying behavior was observed during the measurement of optical resistive behavior of heterostructured films. The studies show how the N_2_ incorporation has considerably altered the energy band alignment at the Ga_2_O_3_/TiO_2_ hetero-interfaces [179]. Thus, the charge transfer mechanisms at Ga_2_O_3_:TiO_2_ and Ga_2_O_3_ (N_2_)/TiO_2_ hetero-interfaces are expected to be different. A bipolar resistive switching performance was observed during *I–V* cyclic test of Au/Ga_2_O_3_–TiO_2_/ITO device under the visible light illumination. The tunable HRS/LRS ratio and loop opening characteristics are the main privileges of Au/Ga_2_O_3_/TiO_2_/ITO optical memristor under the visible light illumination. The Ga_2_O_3_ (N_2_-600 °C)/TiO_2_ heterostructured device has experienced strong rectification behavior which is a characteristic of the development of type-II heterointerface [52, 179, 180]. Thus the main characteristic of this device is its nanoscale ultra-low memristive current, which shows the capability of this unit for the fabrication of the self-rectifying resistive switching-based instruments with low-energy consumption. The nociceptive behavior was observed during the study of reaction of Au–Ga_2_O_3_/TiO_2_–ITO and Au–Ga_2_O_3_ (N_2_-600 °C)/TiO_2_-ITO. The ignition time (*t*_0_) and saturation time (*t*_s_) of the Ga_2_O_3_ (N_2_)/TiO_2_-based nociceptors are shown in Fig. 21a [179]. The relaxation phenomenon is another characteristic of nociceptors, which was observed in the Ga_2_O_3_ (N_2_)/TiO_2_-based heterostructures (Fig. 21b). After the nociceptor ignition the ultimate time for the retrieval of nociceptor to *off* state is called relaxation time. This behavior is similar to the non-adaptation characteristics of the human nociceptors, when the generated warning signals protects the human organs from further unnecessary exposure to hazardous stimuli [180, 181]. It was also found that the relaxation time is the function of the frequency of pulsed light. The abnormal state is a condition when a nociceptor component experiences stimulating signals which are stronger than its natural threshold. In the abnormal state the nociceptor performs similar to the receptor system. Figure 21c shows the difference between the optoresponsive reaction of an abnormal and normal nociceptor. It depicts how the damaged nociceptors show reaction to the constant visible light. The *allodynia* and *hyperalgesia* are the individual characteristics of damaged nociceptor in te abnormal state. The response of nociceptor is continuously increased and the device did not follow the non-adaptation mode when the light intensity is increased (Fig. 21d) after 5 min illumination, which is the characteristic behavior of nociceptors in the abnormal state [179]. Generally, it was found that the nanoengineering and functionalization of semiconductor hetero-interfaces facilitated the fabrication of smart bio-inspired photoreceptors and nociceptor gadgets with self-adapting characteristics.

## Conclusions

The present review has outlined the most recent advancements in the development of wafer-scaled 2D semiconductor nanomaterials for the practical devices fabricated by CVD and ALD techniques. Each of these techniques has its own unique features, merits, complexity, architectures and attractiveness. Despite the rapid progress in the deposition techniques of 2D materials it is fair to say that this field still is in progressing stage. Absolutely new functional opportunities can be initiated and launched if the next generation of advanced functional instruments will be based on 2D semiconductors. Specifically, the outstanding performance of these devices can be achieved through the combinatorial multi-stacking layered 2D nanomaterials. Considering that each of 2D materials has quite different functionalities, multi-functional nanomaterials can be created with enormous advantages in terms of energy and volume. It can be envisaged that a library of 2D materials and nanosheets is required to be updated and renewed regularly. Furthermore, the library of 2D materials is required to be extended by using innovative methods for synthesis of 2D materials from novel material sources.

Moreover, some of the drawbacks of 2D materials fabrication mentioned in review can be solved by obtaining further knowledge about nanointerfaces formation. In this case, the nucleation step and the epitaxial growth has to be controlled in order to avoid or tune eventual interface layer and defects at the interface as well as the mechanical stress and the lattice deformation that can occur in vdW heterostructures. Such stacking imperfections have drastic effects on the physical properties, and therefore, must be addressed for implementation of the final structures. Moreover, the relative rotation angle between both layers directly influences the elastic deformations and hence their tuning permits tailoring the electronic properties of the whole structure. In this regard controlling stack orientation of the ALD fabricated 2D heterostructures would allow the fabrication of 2D materials that have not yet been explored and stipulate new pathways for 2D electronic device fabrication, such as FETs, memristors, resistive switching memory cells and microelectromechanical systems. To enable controlled growth of successive ALD 2D layers, surface functionalization (*chemical* or *physical*) might be required. Despite eventual vdW induced growth, lack of dangling bonds on the 2D surface may inhibit the subsequent deposition. Consequently, the specific functionalization could be realized in order to allow the ALD nucleation while preserving the intrinsic properties of the underlying material.

For further progress in ALD fabrication of various 2D semiconductors, novel ALD precursors must be developed allowing broader expansion of ALD technique in the field of 2D nanomaterials. In this regard, several predicted 2D materials, as silicene, germanene, phosphorene, as well as telluride have not yet been ALD fabricated owing to the lack of suitable precursors. It should also be pointed out that even though graphene is the most studied 2D material, its ALD is still challenging, the only ALD process available leading to material quality inferior to the one obtained using CVD technique.

Finally, one of the main challenges highlighted in many recent published reports is the transition of 2D nanomaterials and their heterostructures into various industrial applications. Currently, only CVD produced graphene is under such transition. Thus, one can envisage that progresses of ALD and CVD of 2D nanomaterials, in particular direct growth of the wafer scale without grain boundaries on integrable devices, will enable their fast industrialization and comercialization.

## Data Availability

Not applicable.

## Abbreviations

ALD: Atomic layer deposition
CVD: Chemical vapor deposition
SEM: Scanning electron microscopy
EDS: Energy-dispersive spectroscopy
XPS: X-ray photoelectron spectroscopy
UV–vis: Ultraviolet–visible
FTIR: Fourier Transform Infrared
PL: Photoluminescence
TMDCs: Transitional metal dichalcogenides
vdW: Van der Waals
COFs: Covalent organic frameworks
FET: Field-effect transistor
HRTEM: High-resolution transmission electron microscopy
VCM: Valence change memristor
ECM: Electrochemical metallization memories
CRS: Complementary resistive switching
MR: Magnetoresistive
RLFP: Random local potential fluctuation
